# Molecular Disambiguation of Heart Rate Control by the Nucleus Ambiguus

**DOI:** 10.1523/JNEUROSCI.0398-26.2026

**Published:** 2026-09-14

**Authors:** Maira Jalil, Mary Katherine MacMillan, Tatiana C. Coverdell, Veronica A. Gutierrez, Maisie E. Crook, Travis J. Heinrichs, Ashlyn Dever, Katie Shouse Cox, Daniel S. Stornetta, Yoko B. Wang, Dana C. Schwalbe, Adriana Singh, Lane VanderVoort, Carie R. Boychuk, Stephen B. G. Abbott, John N. Campbell

**Affiliations:** ^1^Department of Biology, University of Virginia, Charlottesville, Virginia 22904; ^2^Neuroscience Graduate Program, University of Virginia, Charlottesville, Virginia 22903; ^3^Department of Pharmacology, University of Virginia, Charlottesville, Virginia 22903; ^4^Dalton Cardiovascular Research Center, University of Missouri, Columbia, Missouri 65211; ^5^Department of Pathobiology and Integrative Biomedical Sciences, College of Veterinary Medicine, University of Missouri, Columbia, Missouri, 65211

**Keywords:** cardiovagal neurons, diving reflex, heart rate regulation, nucleus ambiguus, parasympathetic nervous system, single-cell RNA-sequencing, vagus nerve

## Abstract

The nucleus ambiguus (nAmb) provides parasympathetic control of cardiorespiratory functions as well as motor control of the upper airways and esophagus. A subset of nAmb neurons innervates the heart through the vagus nerve to control cardiac function at rest and during key autonomic reflexes such as the mammalian diving reflex. Yet, how these cardiovagal nAmb neurons differ from other nAmb neurons in the adult brain remains unclear. We therefore classified adult male and female mouse nAmb neurons molecularly, anatomically, and functionally. First, our integrated analysis of single-nucleus RNA-sequencing data predicted multiple molecular subtypes of nAmb neurons. Mapping the axon projections of one nAmb neuron subtype, *Npy2r*-expressing nAmb neurons, showed that they innervate cardiac ganglia but not the upper airways or esophagus. Chemogenetically stimulating *Npy2r* + nAmb neurons robustly decreased heart rate through peripheral muscarinic acetylcholine receptors. Finally, voluntary underwater diving decreases heart rate and activates *Npy2r* + nAmb neurons, consistent with a cardiovagal function for this nAmb subtype. These results together reveal the molecular organization of nAmb neurons and its control of the heart rate.

## Significance Statement

The nucleus ambiguus (nAmb) of the brainstem controls heart rate through the vagus nerve, but the specific neurons involved have been difficult to identify because they are intermixed with neurons that regulate breathing and swallowing. Here we combine single-cell transcriptomics, anatomical tracing, and physiological experiments to identify a distinct subtype of nAmb neuron, marked by *Npy2r* gene expression, that innervates the heart but not the esophagus or upper airways and powerfully controls heart rate. We also show that these neurons become activated during voluntary underwater diving—a natural reflex that strongly slows the heart. These findings reveal the cellular organization of a key brain–heart circuit and a molecular entry point for studying its role in health and disease.

## Introduction

The parasympathetic nervous system powerfully slows heart rate by signaling through the vagus nerve. This was famously demonstrated in the mid-nineteenth century by the Weber brothers, who found that electrically stimulating the vagus nerve slowed, and in some cases stopped, the hearts of frogs and mammals (Weber, 1846; Fye, 2000). The vagus nerve signals tonically to set resting heart rate and mediates autonomic reflexes such as the baroreflex, respiratory sinus arrhythmia, and mammalian diving reflex (Dampney, 2016; Panneton and Gan, 2020; Ottaviani and Macefield, 2022; Coverdell et al., 2024). The diving reflex, for instance, first described by Edmund Goodwyn in 1786 (Goodwyn, 1786; Vega, 2017), is one of the most robust and evolutionarily conserved of autonomic reflexes (Butler and Jones, 1997; Panneton, 2013; Panneton and Gan, 2020). This reflex occurs when holding breath underwater and triggers a dramatic decrease in heart rate (bradycardia), likely to conserve oxygen stores during an underwater dive (Alboni et al., 2011).

Neurons which innervate the heart through the vagus nerve or cardiovagal neurons (CVNs) reside in the medulla, primarily in the nucleus ambiguus (nAmb) and the dorsal motor nucleus of the vagus (DMV; Geis and Wurster, 1980; Stuesse, 1982; Standish et al., 1994), with the remainder originating in an intervening region known as the intermediate zone (Hopkins and Armour, 1998; Gray et al., 2004). Cardiovagal axons synapse on postganglionic parasympathetic neurons in cardiac ganglia (Cheng et al., 1999; Cheng and Powley, 2000). At these ganglionic synapses, cardiovagal release of acetylcholine (ACh) activates cardiac neurons via α4β2 nicotinic ACh receptors (Li et al., 2009) while potentially suppressing sympathetic input to the same neurons (Levy, 1971). CVNs may release other signaling molecules as well—for instance, neuropeptides such as PACAP (gene, *Adcyap1*) to modulate cardiac neuron excitability (Calupca et al., 2000; Tompkins et al., 2007) and vasoactive intestinal peptide (VIP; gene, *Vip*) to control coronary artery flow (Feliciano and Henning, 1998; Shanks et al., 2023). In turn, cardiac neurons synapse on cardiomyocytes in the sinoatrial (SA) and atrioventricular (AV) nodes, where their release of ACh on muscarinic ACh receptors slows cardiomyocyte electrical conduction and consequently decreases heart rate, AV conduction, and ventricular contractility. Vagotomy or blocking muscarinic receptors increases heart rate, indicating that parasympathetic tone sets heart rate at rest (Reed, 1925; Reed and Layman, 1930; Harvey, 2012).

Previous studies suggest that the nAmb plays a larger role than the DMV in setting heart rate at rest and during reflexes such as the diving reflex (Thomas and Calaresu, 1974; Chen and Chai, 1976; McAllen and Spyer, 1976; Geis and Wurster, 1980; Geis et al., 1981; Panneton et al., 2014). However, the nAmb is a functionally heterogeneous region which, in addition to its CVNs, also contains respiratory parasympathetic neurons and motor neurons for the upper airways and esophagus (Lawn, 1966; Holstege et al., 1983; Fryscak et al., 1984; Bieger and Hopkins, 1987; Coverdell et al., 2024). Recent findings have raised the possibility that nAmb delegates its functional roles to different molecular subtypes of nAmb neurons (Coverdell et al., 2022; Veerakumar et al., 2022; Coverdell et al., 2024). Yet, a comprehensive understanding of these molecular subtypes in the adult brain and their anatomical and functional differences is still lacking. We used single-cell transcriptomics and anatomical tracing to define nAmb neuron subtypes and identified one that innervates cardiac ganglia, decreases heart rate upon activation, and is engaged during the diving reflex.

## Materials and Methods

### Experimental design and statistical analyses

Experimental designs, sample sizes, inclusion/exclusion criteria, and data acquisition procedures are described in the corresponding Materials and Methods subsections, Results, and figure legends, as well as consolidated in Supplemental Table 1. Sample sizes were determined based on previous studies, not on power analyses. Adult male and female mice were used throughout the study, in approximately equal numbers in all experiments unless otherwise indicated. Individual mice were considered biological replicates except for in the snRNA-seq study. Raw and processed snRNA-seq data, metadata, and analysis code will be made openly available through GEO, the Broad Institute Single Cell Portal, and Zenodo as described in the Data Availability section. Experiments were not preregistered.

### Animal subjects

The single-cell RNA-seq experiments used 28–35-week-old male and female B6;129S6-*Chat^tm2(cre)Lowl^*/J mice [“Chat-Cre” (Jackson Laboratory, JAX, strain #028861; Rossi et al., 2011)], some of which had been crossed to a nuclear reporter mouse line B6.Cg-*Gt(ROSA)26Sortm1(CAG-HIST1H2BJ/mCherry,-EGFP/Rpl10a)Evdr*/J (“H2b-TRAP”; JAX strain #029789; Roh et al., 2017). The fluorescent in situ hybridization experiments used C57BL/6J mice from the Jackson Laboratory (JAX, strain #000664). The following mouse lines were used for chemogenetic studies: *Npy2rtm1.1(cre)Lbrl*/RcngJ (“Npy2r-Cre”; gift from Stephen Liberles, Harvard Medical School, Howard Hughes Medical Institute; Chang et al., 2015); Chat^em1(flp)Lowl^ (“Chat-Flp”; gift from Bradford Lowell, Beth Israel Deaconess Medical Center, Harvard Medical School; Coverdell et al., 2022); and R26^ds-HTB^ mice (gift from Martyn Goulding, Salk Institute for Biological Studies). The anterograde tracing experiments used Chat-Cre, Npy2r-Cre, and C57BL/6J mice. Unless otherwise specified, all experiments used adult mice with approximately equal numbers of male and female mice. Mice were housed at 22–24°C with a 12 h light:12 h dark cycle and unlimited access to standard mouse chow and water.

### Fluorescently labeling nAmb neurons

To fluorescently label the nAmb *Chat*+ neurons for single-nuclei transcriptomics, two approaches were used. One approach crossed Chat-Cre mice with an H2b-TRAP transgenic mouse line, which Cre-dependently labels cells with mCherry-tagged histone 2B protein (H2b-mCherry) and an eGFP-tagged ribosomal L10 protein. Preliminary transcriptomic analysis failed to detect *Chat* expression in many Chat-Cre;H2b-TRAP-labeled cells (Fig. 1*A*, far-right panel), consistent with developmental Chat-Cre activity in brain regions around the nAmb. Therefore, a second labeling approach was used to enrich for nAmb *Chat*+ neurons: an AAV which Cre-dependently expresses H2b-mCherry, AAV9-Syn1-FLEX-H2b-mCherry (Vigene Biosciences, 3.94 × 10^13^ GC/ml), injected into the right ventrolateral medulla of eight male and female Chat-Cre mice, 4 weeks prior to tissue collection (see “virus injections” section for details on surgery).

### Single-nuclei RNA-sequencing

Tissue samples of nAmb were collected after rapid decapitation of the mice to avoid stress- and anesthesia-related changes in nuclear mRNA. Brains were immediately extracted, chilled in slush, and sectioned coronally at 1 mm intervals through the nAmb’s full rostral–caudal extent (bregma −6.5 to −8.0 mm). The brain sections were immersed in ice-cold RNAprotect (Qiagen, catalog #76106) to preserve RNA during microdissection and storage. After at least 30 min in RNAprotect, the nAmb was visualized under a fluorescence stereomicroscope (Zeiss Discovery V8), dissected, and stored in RNAprotect overnight at 4°C. On the next day, the nAmb tissue was homogenized and purified by density-gradient centrifugation into a single-nucleus suspension as previously described (Habib et al., 2016; Todd et al., 2020). The single-nucleus suspension was sorted by FACS using 80 nm nozzle to collect H2b-mCherry+ nuclei in a 2 ml microcentrifuge tube. The sample was then processed using 10× Genomics Chromium Next-GEM Single Cell 3′ cDNA Kit v3.1 according to the manufacturer's instructions. The 10× cDNA libraries were sequenced on an Illumina Next-Seq 550 with high-output, 75-cycle v2.5 kits. Sequencing reads were demultiplexed by bcl2fastq2 v2.20.0 (Illumina) and aligned to the mouse genome mm10-2020-A with 10× Genomics Cell Ranger software pipeline 5.0.0 using the include-intron function (Fig. S1*C*).

Gene expression matrices from the 10× Cell Ranger pipeline were input to Seurat v4.3.0 in R (version 4.1.2) for data analysis. Genes detected in fewer than three cells, cells with fewer than 200 genes, and cells with >2% of reads mapping to mitochondrial genes (%mt) were filtered out. Pass-filter data were then merged across batches and integrated to correct for technical differences including batch effects (Stuart et al., 2019). The following steps were then performed: log-normalization of the data; selection of the 2,000 highest-variance genes using Seurat’s FindVariableFeatures() function; batch integration using the IntegrateData() function in Seurat; scaling of gene expression values; principal component analysis (PCA) for linear reduction of the dimensionality of data; cell clustering using Louvain algorithm, based on Euclidean distance in the PCA space comprising the first 13 PCs and with a resolution of 1; and nonlinear dimensionality reduction by Uniform Manifold Approximation and Projection (UMAP) for visualization in two dimensions (Armstrong et al., 2021).

To enrich our analysis with nAmb neurons, we included neuron clusters expressing *Slc18a3*, *Isl1*, and *Chat* and excluded neuron clusters expressing *Slc32a1* and *Slc17a6* (Fig. 1*A*). After removing non-nAmb neurons, the remaining neurons were reclustered, including the steps of feature selection, PCA, clustering with the top 17 PCs and resolution setting of 0.25. Integration of all nAmb neurons was performed again with 12 PCs at 0.25 resolution. Cluster relatedness in PCA space was illustrated with dendrograms using Seurat's BuildClusterTree() function. Differentially expressed genes in each cluster were identified using Seurat's FindAllMarkers() function with the Wilcoxon rank-sum test and *p* values Bonferroni-adjusted for multiple comparisons. The results were filtered to only include positive marker genes expressed in a minimum fraction of 0.25 cells with log_2_-fold change threshold of 0.25. Candidate marker genes were then manually selected based on their expression specificity in each cluster.

### RNA fluorescence in situ hybridization

RNA fluorescence in situ hybridization (RNA FISH) experiments were performed on the brain tissue from mice, some of which received an intraperitoneal injection of 2% Fluorogold (Fluorochrome) to label peripherally projecting neurons, including all nAmb vagal efferent neurons, a minimum of 5 d prior to euthanasia. Mice were terminally anesthetized with ketamine (20 mg/kg) and xylazine (2 mg/kg) diluted in sterile saline, followed by transcardial perfusion with 0.9% saline plus heparin and 4% paraformaldehyde (PFA; Thomas Scientific). Brains were extracted and postfixed in 4% PFA for 24 h at 4°C. Following fixation, brains were sectioned coronally at 30–35 µm thickness on a vibratome (VT-1000S, Leica). The day before FISH, the sections were rinsed in phosphate-buffered saline (PBS) and then mounted on precleaned Superfrost Plus microscope slides (Thermo Fisher Scientific, catalog #12-550-15) and left to dry overnight. An ImmEdge Hydrophobic Barrier Pen was used to draw a barrier around the sections. The sections were then incubated in Protease IV in a HybEZ II Oven for 30 min at 40°C, followed by incubation with target probes (*Chat*, *Phox2b*, *Adcyap1*, *Npy2r*, *Phox2b*, *Cre*, and *Flp*) for 2 h at 40°C. Slides were then treated with AMP 1-3, HRP-C1, HRP-C2, HRP-C3, and HRP Blocker for 15–30 min at 40°C, as previously described (Wang et al., 2012). FITC, Cy3, and Cy5 (Perkin Elmer) fluorophores were used for probe visualization. Fluorogold was imaged based on its native fluorescence. Images were taken using a confocal microscope (Zeiss LSM 800). The three levels of the nAmb correspond to the following bregma levels: rostral or compact nAmb, −6.47 mm through −6.75 mm from bregma; intermediate or semicompact nAmb, −6.83 mm through −7.10 mm from bregma; and caudal or loose nAmb, −7.19 mm through −7.46 mm from bregma.

### Adeno-associated virus injections

Mice were generally anesthetized with ketamine (20 mg/kg) and xylazine (2 mg/kg), positioned in a stereotaxic apparatus (David Kopf Instruments), and locally anesthetized with long-lasting bupivacaine. A pulled glass micropipette was used with a Nanoject III system to stereotaxically inject one of the following adeno-associated viral (AAV) vectors per mouse: AAV9-CAG-FLEX-tdTomato (“AAV9-FLEX-tdTomato”), diluted 1:10 from AddGene stock concentration of ≥1 × 10¹³ viral genomes(vg)/ml, AddGene plasmid #28306, RRID:Addgene_28306, a gift from Edward Boyden; AAV9-CAG-FLEX-PLAP virus (“AAV9-FLEX-PLAP”), diluted 1:10 from stock concentration of ∼1 × 10^13^ genome copies(GC)/ml, a gift from Steve Liberles and Sara Prescott; AAV9-Syn1-DIO-H2b-mCherry (“AAV9-DIO-H2b-mCherry”), Vigene Biosciences, 3.94 × 10^13^ GC/ml; or AAV1/2-CreON/FlpON-hM3Dq-HA, diluted 1:10 from stock concentration of 4.1 × 10^13^ vg/ml, Viral Vector Facility, University of Zurich and ETH Zurich, catalog #vhW34-1. Most AAVs were diluted from stock concentrations to avoid toxicity of an unknown origin which occurred suddenly 2–3 weeks after stereotaxic injections of AAV into the ventrolateral medulla. To span the rostrocaudal extent of the nAmb, multiple injections were made at the following coordinates relative to the calamus scriptorus: anterior/posterior −2.1, −2.4, −2.7 mm, lateral/medial ±1.3 mm, and dorsal/ventral −5.8 mm, from lambda, and anterior/posterior −0.1 mm, lateral/medial ±1.3 mm, and dorsal/ventral −0.1, −1.3 mm. AAV9-FLEX-tdTomato and AAV9-DIO-H2b-mCherry virus was injected unilaterally into the ventrolateral medulla, 50 nl per injection, three injections on the right side per mouse. AAV9-FLEX-PLAP and AAV1/2-CreOn/FlpOn-hM3Dq-HA were injected bilaterally, 50 nl per injection, six injections total per mouse. This injection strategy was designed to fully cover the nAmb, avoid the DMV, and restrict viral infection to only nAmb neurons. The pipette was removed 5 min after each injection, followed by wound closure with sutures or surgical wound glue (Vetbond). Meloxicam SR (5 mg/kg; sustained release) was injected subcutaneously for postoperative analgesia.

### Histology for anterograde tracing studies

Three to four weeks after AAV9-FLEX-tdTomato or AAV9-FLEX-PLAP virus injection, mice were deeply anesthetized with ketamine (20 mg/kg) and xylazine (2 mg/kg) diluted in sterile saline and then transcardially perfused with 0.9% saline plus heparin followed by 4% PFA (Thomas Scientific; CAS#30525-89-4) or 10% neutral buffered formalin (NBF) solution (Sigma-Aldrich HT501128-4L). Brains and hearts were harvested and shaken in the same fixative overnight at room temperature. Brains were either sectioned on vibratome at 35 µm or cryoprotected in 30% sucrose on a shaker at 4°C overnight and then sectioned on freezing microtome at 35 µm. Hearts were cryoprotected in 30% sucrose solution in 4°C on a shaker for 2 d. Hearts were then cut with a 10 blade (Fine Science Tools, catalog #10010-00) along the dorsal ventral axis and were then embedded in optimal cutting temperature compound and sectioned on cryostat (Thermo Fisher Scientific CryoStar NX50) at 10 µm intervals. Slide-mounted cardiac sections were then kept at −80°C for long-term storage.

For heart immunofluorescence, slides were removed from −80°C and placed flat on a tray in a fume hood to dry at room temperature. A hydrophobic barrier was drawn to contain solutions on the slide, and the slides were left to air-dry for 10 min. The slides were then placed in the prechilled Coplin jar containing acetone in −20°C for 10 min. After 10 min, acetone was removed, and the slides dried in the fume hood for 10 min. A 0.1% Sudan Black solution dissolved in 70% ethanol was added for 20 min to reduce autofluorescence. The tissue was then washed three times for 5 min with PBS. The 0.1% PBST was prepared by dissolving 100 ul of Triton X-100 (Sigma Life Sciences, catalog #9036-19-5) in 100 ml 1× PBS (Thermo Fisher Scientific, catalog #70-013-032). Chat-Cre and Npy2r-Cre heart tissues were incubated in a blocking buffer for an hour on a shaker at room temperature. The blocking buffer consisted of 5% donkey serum (Millipore Sigma, catalog #S30-100ML) in 0.1% PBST. For anterograde tracing with AAV-tdTomato, the tissue was incubated overnight at 4°C on a shaker with primary antibodies against PGP9.5 (rabbit, Abcam, catalog #EPR4118) and tdTomato (goat, arigo Biolaboratories, catalog #ARG55724), both diluted 1:500 in 5% donkey blocking buffer. Similarly, to validate brain targeting of AAV-PLAP, brain tissue sections were incubated with anti-PLAP (rabbit, Abcam, catalog #AB133602) and anti-ChAT (goat, Sigma-Aldrich, catalog #AB144P), diluted 1:500 and 1:100, respectively, in 5% donkey blocking buffer.

For brain immunohistochemistry, the tissue was rinsed in PBS three times for 5 min on a shaker at room temperature, followed by incubation in 5% blocking buffer (5% normal donkey serum + 0.1% PBST) for 1 h at room temperature on the shaker. Brain sections were then incubated in primary antibody against ChAT (from goat; Sigma-Aldrich, catalog #AB144P; 1:100 dilution), PLAP (from rabbit, catalog #AB133602; 1:500 dilution), and anti-HA (from chicken, catalog #ET-HA100; dilution 1:500).

After primary antibody incubation, heart and brain sections were then rinsed three times for 5 min in 0.1% PBST solution. Species-specific donkey secondary antibodies conjugated to Alexa Fluor 488, 568, or 647 were obtained from Abcam or Invitrogen, and heart and brain sections were incubated in secondary antibodies (dilution 1:1,000) for 2–4 h on a shaker at room temperature. After three 5 min in PBS washes, the tissue was mounted on SuperFrost Plus precleaned slides (Thermo Fisher Scientific; catalog #12-550-15) followed by coverslipping with Prolong Gold antifade mounting media (Cell Signaling Technology, catalog #9071S) and sealed with nail polish.

### Retrograde labeling of heart-projecting neurons

Mice were deeply anesthetized using isoflurane (2.5%) and placed in a supine position on a heating pad to maintain body temperature. A thoracotomy was performed by making a small incision between the second and third ribs of the right rib cages to expose the heart. A 40 ul volume of Fluorogold (1%; Fluorochrome) was injected into the pericardiac fat pad near the posterior right of AV junction where cardiovagal nerve endings terminate. A small cotton swab was used to absorb any excess tracer to minimize nonspecific labeling. Injection sites were examined after to confirm tracer localization within the pericardiac sac. The incision was closed using suture and animals were returned to their home cage for recovery after surgery and monitored for 3 d. Carprofen (10 mg/kg) and buprenorphine (0.1 mg/kg) were administered immediately after surgery and 1 d postsurgery.

After 1 week, animals were perfused with ketamine (20 mg/kg) and xylazine (2 mg/kg) diluted in sterile saline, followed by transcardial perfusion with 0.9% saline and heparin then 10% NBF solution (Sigma-Aldrich HT501128-4L). The brains were collected and postfixed for 24 h at room temperature in 10% NBF. Following fixation, brains were transferred to 30% sucrose solution for 48 h at 4°C on a shaker and sectioned coronally at 30 or 35 µm thickness on a microtome (SM2010R, Leica Biosystems) for immunofluorescence and RNA FISH as described in the corresponding sections above.

### Placental alkaline phosphatase staining

Following 3–4 weeks after AAV9-FLEX-PLAP virus injection (Prescott et al., 2020), mice were terminally anesthetized with ketamine (20 mg/kg) and xylazine (2 mg/kg) diluted in sterile saline, followed by transcardial perfusion with 0.9% saline plus heparin then 10% NBF solution (Sigma-Aldrich HT501128-4L). The brains and en bloc tissue samples containing the esophagus, trachea, larynx, and pharynx were collected and postfixed for 24 h at 4°C in 10% NBF. Following fixation, brains were transferred to 30% sucrose solution for 48 h at 4°C on a shaker and sectioned coronally at 30 µm or 35 µm thickness on a microtome (SM2010R, Leica Biosystems). A single series of brain sections per mouse was used in histological studies to confirm injection site and PLAP expression. All mice with little to no reporter expression in the nAmb were determined to be surgical “misses,” and so they were excluded from anatomical analyses.

En bloc tissue samples of the esophagus and upper airways were washed three times for 1 h at room temperature in PBS, followed by incubation in alkaline phosphatase (AP) buffer (0.1 M Tris HCl, 0.1 M NaCl, 50 mM MgCl_2_, 0.1% Tween 20, 5 mM tetramisole-HCl), pH 9.5, for 2 h at 70°C. Afterward, the samples were equilibrated to room temperature and then washed twice in AP buffer. AP activity was visualized with NCT/BCIP solution (Thermo Fisher Scientific 34042), and stained samples were rinsed in AP buffer for 15 min, postfixed in 10% NBF for 1 h, and washed in PBS. Samples were then dehydrated through a series of ethanol washes (15–100%) and cleared using a 1:2 mixture of benzyl alcohol (Sigma-Aldrich 402834-500ML) and benzyl benzoate (Sigma-Aldrich B6630-1L). Whole mount images were taken using a bright-field stereomicroscope (Leica M205 FCA Stereomicroscope with color camera), and brain sections were imaged using fluorescence microscope (Echo Revolve).

### Chemogenetic studies with noninvasive electrocardiography and histology

AAV1/2-CreON/FlpON-hM3dq-HA was injected into the bilateral nAmb of Npy2r-Cre;Chat-Flp;R26ds-HTB mice as described above. The mice were allowed to recover for 6–8 weeks before further experimentation. A noninvasive electrocardiography (ECG) system (ECGenie) was then used to measure heart rate. All experiments were performed at the same time of day to avoid circadian differences in heart rate. Mice were acclimated to the ECGenie recording towers for 3 d prior to data collection. Deschloroclozapine (DCZ; Tocris Bioscience, catalog #7193) was prepared by dissolving it in DMSO, then diluting with 0.9% sterile saline to obtain a 2% DMSO solution of DCZ. DCZ-vehicle was prepared by dissolving 2% DMSO in 0.9% sterile saline. After the acclimation period, mice were placed into the ECGenie recording towers for a 10 min baseline recording of heart rate using Corvita data acquisition software (Mouse Specifics, version 2.09). Afterward, DCZ-vehicle was administered by intraperitoneal injection, and heart rate was recorded for 20 min. DCZ was then injected (5 mg/kg, i.p.), and heart rate was recorded for 60 min. Next, volume-matched injections of either vehicle (0.9% sterile saline) or methylatropine (MA) bromide (1 mg/kg, dissolved in 0.9% sterile saline) were administered, and heart rate was recorded for 30 min. Each mouse underwent at least one trial in which MA was injected after DCZ and at least one trial in which MA-vehicle was injected after DCZ. Data were analyzed using the EzCG Analysis Software (Mouse Specifics, version 17). For all ECGenie heart rate recordings, 3–5 s of ECG data were manually selected based on their lack of high-amplitude noise, motion artifact, or dropout and recorded every 3 min. Afterward, ECG signals were visually inspected, and if needed, noisy segments were manually removed, and the signal was inverted if needed, to ensure proper beat detection.

For statistical analyses, heart rate values were averaged per animal for each condition. Comparisons between baseline, vehicle, and DCZ conditions were performed using one-way repeated-measures ANOVA, followed by Tukey's honestly significant difference test for post hoc pairwise comparisons using the emmeans package in R (version 4.1.2), which computes exact Tukey-adjusted *p* values. For the comparisons of MA versus MA-vehicle conditions, paired *t* tests were performed. A *p* < 0.05 was considered statistically significant.

To validate expression of hM3Dq, mice were killed, transcardially perfused, and fixed as described above after completion of the ECG studies. Brains were fixed overnight, transferred to 30% sucrose solution for 48 h at 4°C on a shaker, and sectioned coronally at 30 or 35 µm thickness on a microtome (SM2010R, Leica Biosystems). Sections were rinsed three times for 5 min in PBS and then incubated in blocking buffer (5% normal donkey serum + 0.1% PBST) for 1 h on a shaker at room temperature. After blocking, sections were incubated overnight at 4°C on a shaker in primary antibody solution containing goat anti-Chat (primary antibody at 1:100 dilution in blocking buffer, Sigma-Aldrich catalog #AB144P, lot #3491643) and chicken anti-HA antibody (1:500 dilution, Aves Labs, lot #HA7757994, catalog #ET-HA 100). The following day, sections were rinsed three times for 5 min in 0.1% PBST solution (100 ul Triton X-100 dissolved in 100 ml sterile PBS) on a shaker and then incubated in secondary antibody for 2–4 h at room temperature. Donkey anti-chicken Alexa Fluor 647 (Jackson ImmunoResearch Laboratories, reference #703-585-155, lot #147524; 1:1,000 in 0.1% PBST) and donkey anti-goat, Fluorophore 488 (Invitrogen, reference #A11055, lot #2747580; 1:1,000 in 0.1% PBST). Sections were then rinsed in PBS three times for 5 min on a shaker at room temperature. After rinsing, sections were mounted onto slides, allowed to dry, and coverslipped using VECTASHIELD Antifade Mounting Medium with DAPI (catalog #H-1200-10) and then sealed with nail polish. Slides were imaged using the confocal microscope. Mice were excluded from functional analysis if their nAmb lacked hM3Dq-HA immunofluorescence or their DMV contained >5 hM3Dq-HA immunofluorescent neurons.

### Dose–response curve

Before the dose–response curve experiments, mice were acclimated to the noninvasive ECG towers (ECGenie, Mouse Specifics) for one 30 min session per day for 3 d. On experiment days, mice were first acclimated to the towers for 15 min before being placed in their home cage for 15 min. Then, for baseline recordings, mice were placed in the ECG towers for 15 min, and one 3–8 s ECG signal was recorded every 5 min, for a total of three recordings. These three recordings were averaged for each mouse (baseline data point). Then, mice were injected intraperitoneally with the lowest dose of DCZ (0.01 mg/kg; Tocris Bioscience, catalog #7193, dissolved in 2% DMSO in 0.9% sterile saline) and placed back into their home cages. After 15 min, mice were again placed in the towers for 15 min, where one ECG measurement was collected every 5 min, for a total of three recordings per dose. This was repeated for subsequent DCZ doses. A cumulative design was used so that each dose given was added to the previous dose (e.g. after the 0.01 mg/kg dose, mice are injected with 0.09 mg/kg to reach the 0.1 mg/kg dose). This experiment was repeated on a nonconsecutive day using 0.9% sterile saline for all injection doses.

For ECG analysis, an average of 3–8 s of data was collected for each measurement, with three measurements per dose per mouse. This system requires that data are visualized and manually selected during collection, so that only data free of technical artifacts (no high-amplitude noise or dropout) were collected and subsequently analyzed. ECG data were excluded solely based on technical artifacts such as motion or insufficient contact with the ECG electrode.

### Voluntary underwater dive training

Npy2r-Cre;Chat-Flp;dsHTB and C57Bl/6J mice were trained to voluntarily dive underwater according to a protocol adapted from a previous publication (Hult et al., 2019). A diving pool was built from a 69-cm-long × 38-cm-wide × 10-cm-high plastic tub. A channel was created in the center of the pool by affixing two 69-cm-long × 0.32-cm-wide × 10-cm-high plexiglass strips with 2.5 cm perpendicular ledges 6 cm from the bottom, one-third and two-thirds of the way across the length of the pool. These served as rails along which water-level barriers were placed to extend the length of the underwater dive. Squares of 10 × 0.6 × 10 cm plexiglass were used to construct walls around the starting end of the pool. To set up the diving enclosure for training, the two plexiglass strips were taped in place onto either end of the container and water was added to the tank to the desired level. Water temperatures were kept between 33 and 37°C to prevent hypothermia and minimize stress. A raised platform was placed at the opposite end for the mouse to climb out of the water (escape platform). Small circles were drawn every 15 cm on the inside walls of the two plexiglass strips to help guide the mice while underwater. Additionally, a large “X” was drawn on the underwater portion of the platform for the same purpose. The diving enclosure was set up with the platform and thermometer and filled with water before every dive training session and drained and cleaned after every session. A heat lamp was set up ∼30 cm above the recovery cages to provide warmth after dive completion. At the start of each swim or dive, the mouse was taken from its home cage, placed at the starting end of the pool, allowed to swim across to the escape platform, and then removed from the pool and placed into its home cage under the warming lamp.

A 5 d progressive protocol for dive training mice was performed as follows. On Day 1 of dive training, mice were allowed 2 min to explore the pool with 1 cm of water. If a mouse touched the escape platform with all four paws within 2 min, they were immediately removed from the pool. If they failed to reach the escape platform, they were removed after 2 min. This was repeated seven times. On Day 2 of dive training, mice were allowed to explore with the same criteria as in Day 1, for two trials. Then, water was added up to 3 cm, and mice were allowed to wade through the water, for two trials. Water was added up to 5 cm, so the mice were unable to touch the bottom of the enclosure while swimming, for seven trials. On Day 3, mice underwent a series of underwater dive trials of increasing distances: one trial of a 1-cm-long dive; two trials of a 3-cm-long dive; four trials of a 5-cm-long dive; and six trials of a 6-cm-long dive. On Day 4, mice underwent one trial each of 1-, 3-, and 5-cm-long dives, two trials of a 1-cm-long dive, and five trials of a 10-cm-long dive. On Day 5, mice did one trial each of 1-, 3-, and 5-cm-long dives, two trials each of a 1- and a 10-cm-long dive, and five trials of a 20-cm-long dive. Dive repetitions were increased or decreased depending on each mouse’s learning speed.

### Instrumentation for telemetric ECG

Mice used for the diving reflex studies and a subset of mice used for the chemogenetic studies underwent surgical implantation of ECG telemeters (Data Science International, DSI; ETA-F10) for wirelessly recording heart rate. Each mouse was anesthetized with isoflurane and placed in dorsal recumbency on the autoheated surgical table. The skin on the chest and upper abdomen was shaved and cleaned with alcohol and iodine, and 0.1 ml of Nocita (long-lasting bupivacaine, 13.3 mg/ml) was injected at the midline of the sternum, with the limbs loosely taped down. A 2 cm shallow, vertical inline incision was made along the sternum and, using blunt-tip hemostatic forceps, a subcutaneous pocket starting at the left caudal edge of the incision was created down the lateral flank. Once the pocket size was increased to ensure adequate fit for the probe, the pocket and the probe were rinsed with sterile saline. The device ID number was noted alongside the mouse ID, and the probe was inserted into the pocket with the leads oriented cranially (red lead on the left and white lead on the right). The leads were trimmed to the appropriate length to allow for placement in desired locations. The red lead was routed subcutaneously by blunt dissection to the left flank inferior to the underarm and secured with a 6.0 silk suture to the muscle. The white lead was routed subcutaneously across the torso to the upper-right pectoralis major and secured with a 6.0 silk suture. Secure attachment of the probe was confirmed by slightly tugging on the leads. The incision was closed with three mouse wound clips, and antibiotic ointment was applied. The wound clips were reapplied if dislodged by the mouse. Wound clips were subsequently removed 7–10 d later.

### Telemetric ECG

For long-term, continuous heart rate recordings in response to chemogenetic activation of Npy2r+ neurons with DCZ, mice were implanted with implantable telemetry ECG probes (DSI; ETA-F10) for recording heart rate wirelessly. After recovery from the implant, mice were acclimated to the behavioral room, DSI system, and injection paradigm for 4 h per day for 3 consecutive days. During acclimation and recording, mice in their home cages were placed on the DSI recording pads, with *ad libitum* access to food and water. After 55 min, mice were administered vehicle injections (2% DMSO in 0.9% sterile saline) and placed back into their home cages for the remainder of recording.

On experimental days, mice were brought upstairs into the testing room between 11:30 P.M. and 12:00 P.M. and allowed to acclimate for 15 min to account of any stress. Then, ECG probes were turned on, and ECG was collected at 1,000 Hz for the remainder of the recording. After 55 min of baseline recording, mice were removed from their home cage, intraperitoneally injected with 5 mg/kg DCZ (Tocris Bioscience, catalog #7193), dissolved in 2% DMSO and 0.9% sterile saline or vehicle (2% DMSO in 0.9% sterile saline), and returned to their home cage as the recording continued for 3 h postinjections. Afterward, DSI probes were turned off, and mice were returned to the colony. The same mice underwent DCZ and vehicle trials on nonconsecutive days.

ECG data were analyzed offline using DSI’s Ponemah Software Electrocardiogram Analysis Module (v6.5). First, data were manually inspected for large periods of dropout or technical noise, and those sections of data were excluded manually using the “Bad Data Marks” tool. Approximately 6% of data were excluded because of such technical artifacts. Afterward, all data were manually inspected, and R-peak detection parameters were adjusted to improve beat detection, including QRS detection threshold and Min R Deflection. If needed, individual R-peaks were manually added. The logging rate was set to average data within 5 min windows, and these data were exported into a Microsoft Excel document for later analysis with R software.

### Diving reflex assay and histology

For diving-induced *Fos* experiments, a total of 13 mice (seven females, six males) were trained according to the above protocol. Before training, mice were administered Fluorogold systemically (FG; intraperitoneal as described above) to label nAmb neurons. After 5–7 d of training, mice were randomly assigned into groups (voluntary dive or no dive). For voluntary diving, seven mice (four females, three males) were introduced into the diving chamber filled with water and allowed to dive voluntarily to reach the platform. Upon reaching the platform, mice were given 10–15 s rest before being returned to the opposite end of the apparatus to dive again. For 20–30 min, mice completed 20–40 individual dives before being placed in a warmed recovery chamber for 20 min to allow time for *Fos* mRNA expression. As a control (“no dive”), mice were placed in the diving enclosure filled with 1 cm of water and allowed to explore for 30 min before being placed in a warmed recovery chamber for 20 min before perfusion.

Mice were deeply anesthetized with a mixture of ketamine (100 mg/kg) and dexmedetomidine (0.2 mg/kg) given intraperitoneally and perfused transcardially with 4% PFA, pH 7.4, in 100 mM phosphate buffers. Brains were removed and postfixed in the same fixative for 12–24 h at 4°C. Brains were sectioned (30–50 µm) on a vibratome (VT-1000S, Leica Biosystems), and sections were stored in cryoprotectant [30% ethylene glycol (v/v), 20% glycerol (v/v), 50% 100 mM phosphate buffer], pH 7.4, at −20°C. Multiplex fluorescent in situ hybridization was performed using RNAscope (V1 kit, Advanced Cell Diagnostics). Probes for *Npy2r*, *Chat*, and *Fos* and native fluorescence were used to identify Fluorogold+ cells. Serial one-in-three sections were washed in RNase(ribonuclease)-free PBS, mounted on charged slides, dried overnight, and processed according to the manufacturer's instructions. Immediately following the RNAscope procedure, sections were rinsed and then incubated in blocking solution for 10 min followed by incubation in primary at room temperature for 60 min, rinsed and incubated in secondary antibodies for 30 min, and rinsed and then dried overnight before coverslipping. Slides were coverslipped with ProLong Gold antifade mounting media with DAPI (Thermo Fisher Scientific, catalog #P3693).

All nAmb neurons on one side of the brain between the spinal decussation and facial nucleus were counted. Only cells that contained FG fluorescence and stained for *Chat* RNA were included. The presence or absence of *Npy2r* and *Fos* RNA was determined for each FG+/*Chat* + nAmb neuron. A cell was considered *Fos*+ if it contained >5 bright *Fos* puncta.

## Results

### Molecular classification of nAmb neurons in the adult mouse

To classify molecular subtypes of nAmb neurons in adult mice, we compared their genome-wide RNA expression using single-nuclei RNA-sequencing (snRNA-seq). First, to enrich for nAmb neurons, we fluorescently labeled their cell nuclei based on expression of the cholinergic marker gene, *Chat*, which encodes choline acetyltransferase (ChAT) and which all nAmb vagal efferents and few neighboring cells express (Coverdell et al., 2022; Fig. S1*A*). Specifically, we crossed mice in which Cre recombinase expression is driven by the *Chat* gene (Chat-Cre; Rossi et al., 2011), to mice which Cre-dependently express a nuclear-localized mCherry fluorescent protein (H2b-mCherry; “H2b-TRAP” mice; Roh et al., 2017). The resulting Chat-Cre;H2b-TRAP mice exhibited H2b-mCherry expression in all peripherally projecting nAmb neurons (Coverdell et al., 2022; Fig. S1*B*). As a complementary approach, to avoid any cells labeled due to Chat-Cre expression only during development, we injected the nAmb of other adult Chat-Cre mice with an AAV that Cre-dependently expresses H2b-mCherry. We then isolated mCherry+ cell nuclei from dissected nAmb tissue samples, processed their poly-adenylated RNA into sequencing libraries, and sequenced them (Fig. 1*A*).

**Figure 1.**
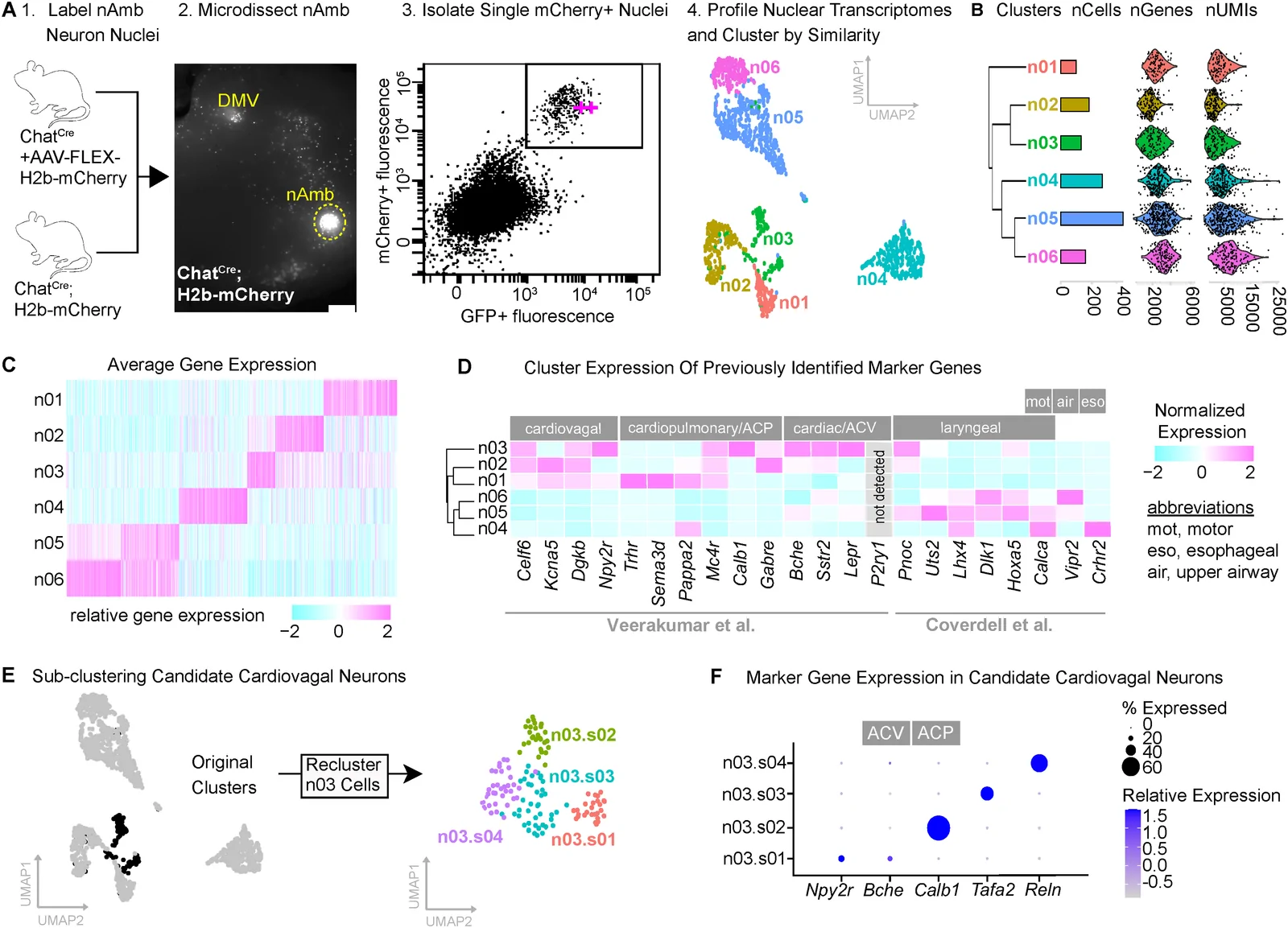
Molecular classification of vagal efferent neurons in the ventrolateral medulla of the adult mouse. ***A***, Schematic of the snRNA-sequencing workflow. From left to right: labeling and isolation of nAmb neurons using Chat-Cre;H2b-TRAP mice and Chat-Cre mice injected with a Cre-dependent AAV-H2b-mCherry; selection of mCherry+/GFP + nuclei from Chat-Cre;H2b-TRAP mice during fluorescence activated nuclei sorting; UMAP of ventrolateral medulla vagal neuron clusters. ***B***, From left to right, dendrogram illustrating relatedness of clusters; number of cells, genes and unique molecular identifiers (UMIs; ∼unique RNA transcripts). ***C***, All genes differentially expressed among clusters to a statistically significant extent based on a Bonferroni-adjusted *p* < 0.05. ***D***, nAmb neuron cluster expression of subtype marker genes reported in previously published studies. ***E***, Subclustering cells from candidate cardiovagal cluster n03. ***F***, Expression of candidate marker genes for n03 subclusters. ACP and ACV marker genes labeled based on Veerakumar et al.

Our initial dataset contained 3,490 nuclear transcriptomes (“cells”) across three batches (Fig. S1*C*). Filtering out low-quality transcriptomes and likely cell doublets left 3,461 cells for further analysis. Grouping the pass-filter cells by their expression of high-variance genes yielded 18 clusters (annotated as c1-c18), each containing cells from each sample batch (Fig. S1*D*–*G*). To identify clusters of vagal efferent neurons, we plotted expression of positive marker genes associated with cholinergic and motor neurons (vAChT/*Slc18a3*, *Isl1*, *Chat*) and negative marker genes (*Slc32a1*, *Slc17a6*) which are expressed in nearby, nonvagal neurons (Tanaka et al., 2003; Sherman et al., 2015; Anderson et al., 2016; Coverdell et al., 2022; Fig. S1*H*). Consistent with these marker expression patterns, our filtered dataset contained only vagal efferent neurons from the ventrolateral medulla, not vagal afferent neurons from the nodose ganglia or other noncholinergic neurons. Based on our results, we annotated five clusters, containing 1,245 neurons in total, as candidate subtypes of vagal efferent neurons in the ventrolateral medulla (Fig. S1*H*).

We further explored the molecular heterogeneity these vagal efferent neurons by reclustering them apart from the other neurons in our initial dataset. This resulted in six transcriptionally distinct clusters (n01-n06) in which we detected on average (±standard deviation, SD) 2,927 ± 967 genes per neuron, based on 6,666 ± 3,565 unique transcripts per neuron (Fig. 1*B*). Each cluster contained cells from each sample batch (Fig. S1*I*,*J*). Comparing the neuron clusters revealed many differentially expressed genes (Fig. 1*C*; Fig. S1*K*). Among these was the gene *Adcyap1*, which is enriched in clusters n02 and n03 (Fig. S1*K*) and encodes pituitary adenylate cyclase activating polypeptide (PACAP), a neuropeptide found in cholinergic axons innervating the heart in guinea pigs (Calupca et al., 2000). Based on these results, we hypothesize that clusters n02 and n03, which are adjacent clusters on the UMAP but transcriptionally distinct (Fig. 1*C*), may contain CVNs.

To assess the robustness of the candidate cardiovagal clusters, we evaluated their stability across a range of clustering parameters. Cells from all three sequencing batches contributed to each cardiovagal cluster (n01–n03), indicating that these populations were not derived from a single sequencing batch (Fig. S2*A*,*B*). We next evaluated cluster stability across a range of Louvain clustering resolutions using Clustree (Zappia and Oshlack, 2018; Fig. S2*C*) and observed that the candidate cardiovagal clusters were stable across multiple resolutions, with each cluster maintaining a distinct lineage. Similar cluster organization was observed when the data were reclustered using different numbers of principal components (10, 14, and 16 PCs; Fig. S2*D*). Finally, we performed a leave-one-batch-out analysis by independently excluding either sample Batch 1 or 2 and repeating the clustering analysis. Both approaches recovered all major neuronal clusters and their corresponding marker gene expression patterns (Fig. S2*E*,*F*). These results indicate that our clusters of vagal efferent neurons, including those of candidate CVNs, are stable across a range of clustering parameters.

We then examined cluster-level expression of marker genes for nAmb neuron subtypes described by previous transcriptomic studies. One of these studies, Coverdell et al. (2022), identified three molecular subtypes among 141 adult mouse nAmb neurons: Crhr2^nAmb^ neurons, which control esophageal motor function; Vipr2^nAmb^ neurons, which innervate the pharynx and larynx; and Adcyap1^nAmb^ neurons, whose anatomy and function were unknown (Coverdell et al., 2022). Another study, Veerakumar et al. (2022), characterized three molecular subtypes from 203 neonatal mouse nAmb neurons innervating the larynx or heart: ACP neurons and ACV neurons, which can alter cardiopulmonary and cardiovascular function, respectively, and larynx-projecting neurons (Veerakumar et al., 2022). When we plotted the expression of these published marker genes in our dataset, we found that clusters n01, n02, and n03 showed marker gene expression most consistent with cardiovagal parasympathetic motor neurons, whereas clusters n04, n05, and n06 transcriptomic profiles aligned to branchial motor neurons that innervate skeletal muscles of the upper airway and esophagus (Fig. 1*D*).

Cluster n03 showed relatively high expression of marker genes associated with two distinct, previously defined subtypes of CVNs, ACP neurons and ACV neurons (Fig. 1*D*; Veerakumar et al., 2022), suggesting heterogeneity within cluster n03. Reclustering only the neurons from n03 divided them into four groups or “subclusters” (n03.s01–04; Fig. 1*E*). One of these subclusters, n03.s01, specifically expressed a gene marking ACV neurons (*Bche*), while another, n03.s02, specifically expressed a gene marking ACP neurons (*Calb1*; Fig. 1*F*), consistent with previous descriptions of these subtypes (Veerakumar et al., 2022). Interestingly, the putative ACV neurons also expressed *Npy2r*, the gene encoding the neuropeptide Y2 receptor, which modulates vagal control of heart rate (Herring et al., 2008) and is expressed by vagal sensory neurons that control heart rate (Smith-White et al., 2002; Herring et al., 2008; Chang et al., 2015). Together, our results identify nine candidate molecular subtypes of vagal efferent neurons in the ventrolateral medulla, including six candidate cardiovagal subtypes, and raise the possibility that *Npy2r^+^* nAmb neurons contribute to heart rate control.

To compare in a less biased manner our candidate subtypes of vagal efferent neurons to those identified by previous studies, we used reference query mapping. This method projects query cell transcriptomes onto reference cell clusters without altering the reference data structure (Hao et al., 2021). Interestingly, while the Coverdell et al. cell clusters largely showed the highest correspondence to the cell clusters of the present dataset (Fig. S3*A*), the Veerakumar et al. cells subdivided among the same three reference clusters (Fig. S3*B*). When we assessed the confidence of cell mapping, most Coverdell et al. cells mapped with higher confidence to the reference clusters (>0.8; Fig. S3*C*) than did the Veerakumar et al. cells (<0.6; Fig. S3*D*). This suggests that the adult nAmb neurons of Coverdell et al. are transcriptionally more similar to the adult nAmb neurons in our present dataset than are the neonatal nAmb neurons of Veerakumar et al., which could be due to developmental differences in gene expression. In line with this possibility, the larynx-projecting neurons of Veerakumar et al. but not in those of Coverdell et al. (Fig. S3*E*,*F*) expressed the gene *Adcyap1*, consistent with its broader expression in the developing brain (Shuto et al., 1996). Thus, the ability to match neonatal and adult nAmb neurons with the reference query approach may be limited by developmental differences in gene expression.

### Partially overlapping populations of nAmb neurons express *Npy2r* and *Adcyap1*

Given the possibility that CVNs could express *Npy2r* and *Adcyap1*, we visualized their transcripts in the adult mouse nAmb using RNA FISH. First, to label all peripherally projecting neurons, including all vagal efferent neurons, we intraperitoneally injected adult C57BL6/j mice with the retrograde tracer Fluorogold (Schmued and Fallon, 1986; Merchenthaler, 1991). We then performed RNA FISH for *Adcyap1*, *Npy2r*, and *Chat* transcripts in coronal sections and imaged them throughout the nAmb's compact, semicompact, and loose subregions. We observed *Npy2r* + nAmb vagal efferent neurons scattered throughout these subregions (Fig. 2*A*, representative images), consistent with the known anatomical distribution of cardiovagal nAmb neurons (Geis et al., 1981; Kalia, 1981; Stuesse, 1982; Veerakumar et al., 2022). Quantifying nAmb neurons expressing *Adcyap1* and/or *Npy2r* revealed a partially overlapping distribution. Specifically, 4.0% of nAmb neurons expressed both *Npy2r* and *Adcyap1*, 3.0% expressed *Npy2r* but not *Adcyap1*, and <0.3% expressed *Adcyap1* but not *Npy2r* (Fig. 2*A*,*B*). Thus, only a small minority of nAmb vagal efferent neurons expressed *Adcyap1* and/or *Npy2r*. Of note, the 8% of nAmb neurons expressing *Adcyap1* and/or *Npy2r*, extrapolated for the total number of adult mouse nAmb neurons (Sturrock, 1990), would be ∼47 neurons per nAmb, slightly below previous estimates of retrogradely labeled cardiovagal nAmb neurons in mice (50–60 neurons; Veerakumar et al., 2022).

**Figure 2.**
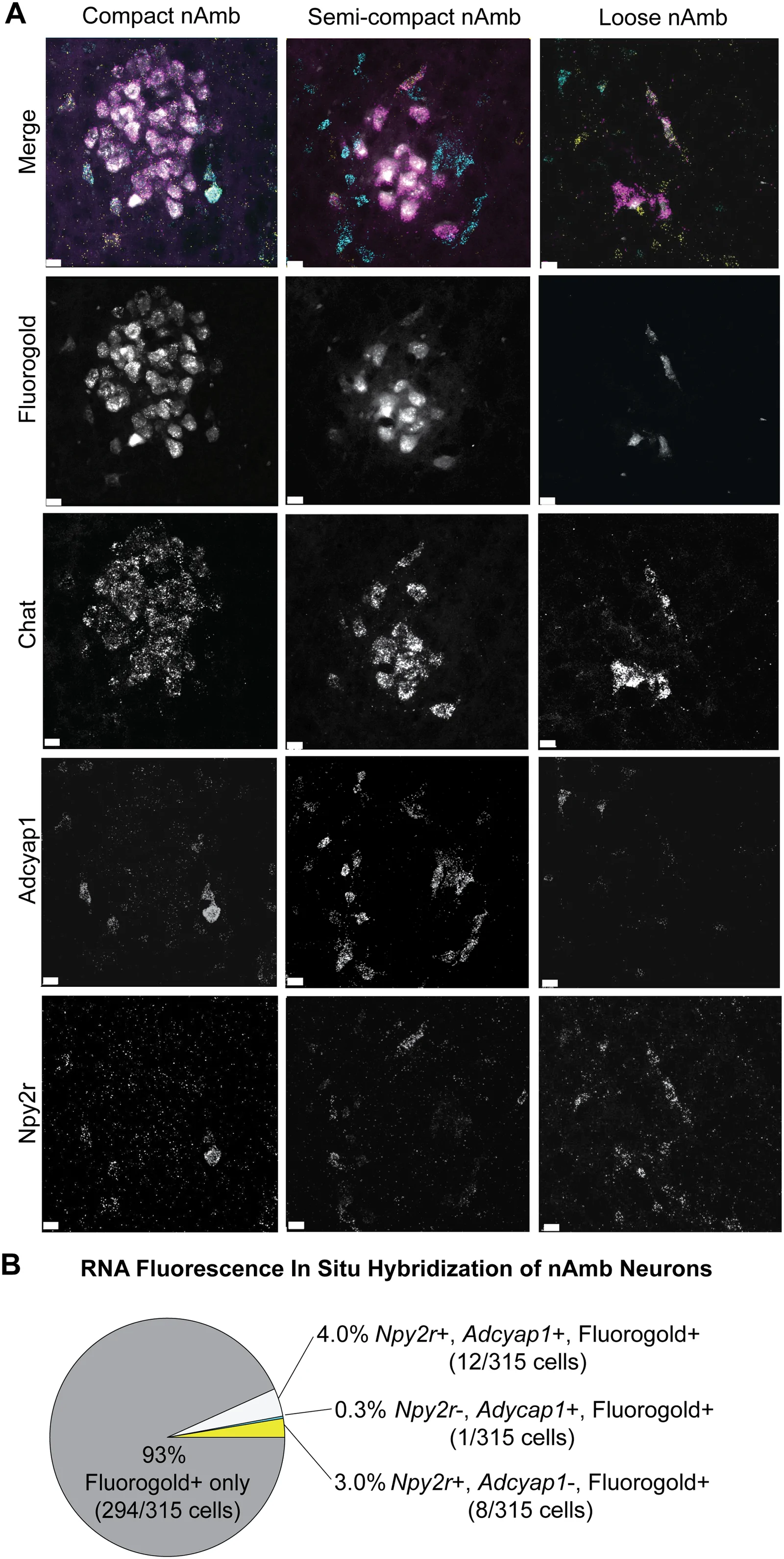
Partially overlapping populations of nAmb neurons express *Npy2r* and *Adcyap1*. ***A***, RNAscope fluorescent in situ hybridization (RNA FISH) for *Npy2r* and *Adcyap1* in compact, semicompact, and loose subdivisions of the nAmb. All nAmb vagal efferent neurons were identified by RNA FISH for *Chat* and systemic Fluorogold labeling (scale bar, 20 μm). All individual color channels are displayed in grayscale. In the merged image, Fluorogold is shown in gray, *Chat* in magenta, *Adcyap1* in cyan, and *Npy2r* in yellow. ***B***, Quantification of RNA FISH data (*n* = 3 mice).

### Retrograde labeling from the heart reveals CVN subtypes in the nAmb and intermediate zone

To identify heart-projecting vagal motor neurons, we injected the retrograde tracer Fluorogold into the pericardiac fat pad near the posterior right AV junction, where many cardiovagal nerve endings terminate (Strain et al., 2024; Fig. 3*A*). We inspected the injection site for potential tracer leak and to confirm minimal tracer spread (Fig. S4*A*). One week later, we collected brainstem tissue for Fluorogold immunofluorescence and RNA FISH. On average, we identified 40 ± 24 Fluorogold-labeled cells per mouse bilaterally in the nAmb across 17 brain sections from four mice, distributed across the full rostral–caudal extent of the nAmb (Fig. 3*B*). Among these, 27% contained *Npy2r* transcripts by RNA FISH (Fig. 3*C*). These results indicate that *Npy2r* *+* nAmb (Npy2r^nAmb^) neurons are a subset of cardiovagal nAmb neurons.

**Figure 3.**
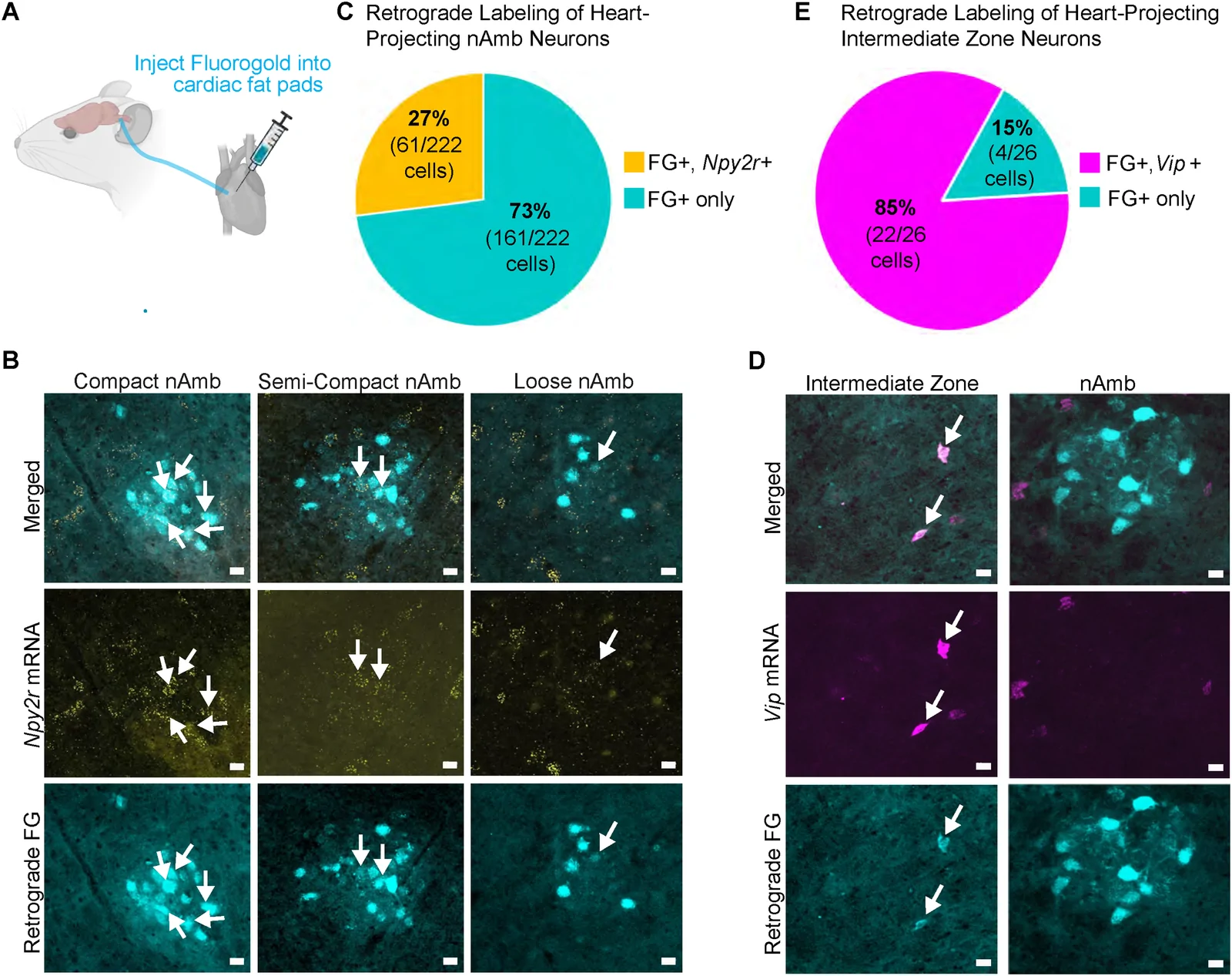
Retrograde labeling from the heart reveals cardiovagal populations in the nAmb and intermediate zone. ***A***, Schematic of retrograde tracing from heart with Fluorogold. ***B***, *Npy2r* RNA FISH and Fluorogold labeling in the compact, semicompact, and loose nAmb. Scale bar, 20 μm (*n* = 4 mice). ***C***, Quantification of *Npy2r*+ cells as a subset of Fluorogold + nAmb cells. ***D***, *Vip* RNA FISH and Fluorogold labeling in the compact, semicompact, and loose nAmb. Scale bar, 20 μm (*n* = 3 mice). ***E***, Quantification of *Vip*+ cells labeled with retrograde Fluorogold in the intermediate zone.

Another neuronal cluster identified in our snRNA-seq analysis, cluster n02 (*Vip*+/*Chat*+/*Phox2b*+), was transcriptionally most similar to *Npy2r* + nAmb neurons (cluster n03; Fig. 1*B*, cladogram), raising the possibility that n02 neurons are also cardiovagal. To test this, we retrogradely labeled heart-projecting neurons by injecting Fluorogold into the pericardiac fat pad, as described above, and assessed colocalization of Fluorogold with RNA FISH for *Vip*, a marker for cluster n02 neurons, in the ventral and lateral medulla. Surprisingly, none of the 221 Fluorogold-labeled nAmb cells examined across three mice contained *Vip* transcripts (Fig. 3*D*). Instead, we observed robust colocalization of *Vip* transcripts with Fluorogold labeling in the intermediate zone between the nAmb and the DMV, where 85% of Fluorogold-labeled cells also contained *Vip* transcripts (Fig. 3*D*,*E*). These findings indicate that the *Vip* *+* neuron cluster (n02) corresponds to CVNs of the intermediate zone rather than the nAmb and that their inclusion in our snRNA-seq most likely reflects a dissection artifact. Of note, the *Vip* gene product, VIP, contributes to parasympathetic cardiac regulation by modulating heart rate, influencing intracardiac ganglion excitability, and interacting with cholinergic signaling mechanisms that generate cardioinhibitory output (Weihe et al., 1984; Henning and Sawmiller, 2001). Together, our retrograde-tracing results demonstrate at least two molecularly and anatomically distinct neuron populations in the ventrolateral medulla innervate the heart: *Npy2r+* neurons in the nAmb and *Vip*+ neurons in the intermediate zone.

### Npy2r^nAmb^ neurons innervate cardiac ganglia

Since retrograde tracers injected into the heart can spread to other visceral tissues and label non-CVNs (Grkovic et al., 2005), we performed cell type-specific anterograde tracing to confirm that Npy2r^nAmb^ neurons innervate the heart. Specifically, we labeled the axons of nAmb neurons with an AAV that Cre-dependently expresses the red fluorescent protein, tdTomato (AAV9-DIO-tdTomato). We confirmed the Cre dependency of AAV9-DIO-tdTomato by injecting it into the ventrolateral medulla of adult C57BL6/j mice, where we failed to observe tdTomato immunofluorescence 3 weeks later at the injection sites (*n* = 3 mice; Fig. S4*B*). Next, to target this AAV to Npy2r^nAmb^ neurons, we obtained Npy2r-Cre mice (Chang et al., 2015). We validated the specificity and sensitivity of Cre expression in these mice by colocalizing *Npy2r* and *Cre* transcripts by RNA FISH in the nAmb. In Npy2r-Cre mice, 92% (145 of 157) *Npy2r* mRNA+ neurons were also *Cre* mRNA+, and 100% (145 of 145) of *Cre* mRNA+ cells were also *Npy2r* mRNA+ (*n* = 3 mice; Fig. S4*C*). These results validate the specificity of AAV9-DIO-tdTomato and Npy2r-Cre mice for genetically targeting Npy2r^nAmb^ neurons.

To fluorescently label Npy2r^nAmb^ axons for anterograde tracing, we injected the ventrolateral medulla of Npy2r-Cre mice with AAV9-DIO-tdTomato (Fig. 4*A*). As a positive control, we also injected the ventrolateral medulla of Chat-Cre mice (Rossi et al., 2011), which express Cre activity in all nAmb vagal efferent neurons (Coverdell et al., 2022; Chat^nAmb^ neurons). After waiting at least 3 weeks for AAV transgene expression, we observed tdTomato immunofluorescence in a subset of nAmb neurons, identified based on their immunofluorescence of the cholinergic marker ChAT. While some cells outside the nAmb in Npy2r-Cre mice were also tdTomato+, none of these were ChAT+ (Fig. 4*B*), indicating that they were not vagal efferents and so would not confound our anterograde tracing results. Importantly, we observed no tdTomato+ neurons in the DMV, another source of heart innervation (Fig. S4*D*; Nosaka et al., 1979; Stuesse, 1982). Thus, our AAV9-DIO-tdTomato injections labeled vagal motor neurons in the nAmb but not the DMV.

**Figure 4.**
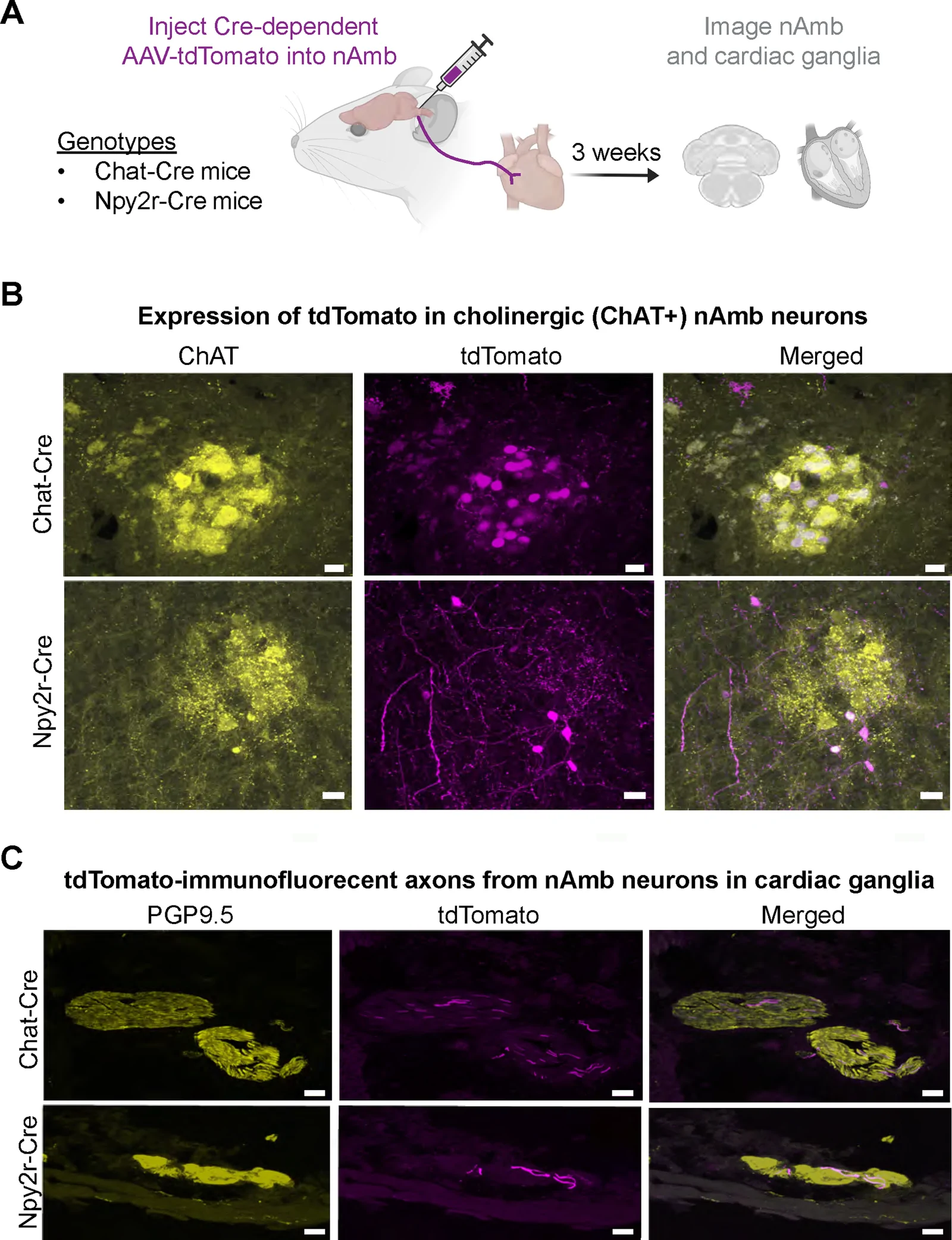
Npy2r^nAmb^ neurons innervate cardiac ganglia. ***A***, AAV9-FLEX-tdTomato injected into right side nAmb of Npy2r-Cre mice and, as positive controls, Chat-Cre mice (*n* = 3 mice per genotype). Figure panel created with Biorender.com. ***B***, tdTomato and ChAT immunofluorescence in nAmb of Chat-Cre mice and Npy2r-Cre mice. Scale bar, 20 µm. ***C***, tdTomato axons of Chat-Cre and Npy2r-Cre nAmb neurons in PGP9.5 immunofluorescent cardiac ganglia. Scale bar, 20 µm.

To determine whether Npy2r^nAmb^ neurons innervate the heart, we then imaged immunofluorescence of tdTomato and the neuronal marker protein PGP9.5 in the cardiac tissue from the same mice. PGP9.5 is expressed generally by neurons, including all cardiac ganglia neurons in rodents (Richardson et al., 2003). We observed fibers immunofluorescent for both tdTomato and PGP9.5 innervating PGP9.5 + cardiac ganglia in all Npy2r-Cre mice as well as in all positive-control Chat-Cre mice (Fig. 4*C*; *n* = 3 mice per genotype; at least two innervated ganglia per mouse). These results indicate that Npy2r^nAmb^ neurons innervate cardiac ganglia, confirming their identity as CVNs.

### Npy2r^nAmb^ neurons do not innervate the upper airways or esophagus

Since the nAmb also contains motor neurons for the esophagus and upper airways (Holstege et al., 1983; Fryscak et al., 1984; Bieger and Hopkins, 1987; Haxhiu et al., 1993; McGovern and Mazzone, 2010), we investigated whether Npy2r^nAmb^ neurons project to those tissues using a chromogenic anterograde tracing approach. First, we injected an AAV encoding Cre-dependent placental AP (AAV9-FLEX-PLAP) into the ventrolateral medulla of Npy2r-Cre mice (*n* = 3 mice). As a positive control, we performed the same injections in Chat-Cre mice (*n* = 3 mice; Fig. 5*A*; Coverdell et al., 2022). At least 3 weeks later, we collected the medulla and en bloc tissue samples of the esophagus and upper airways for histology. We observed PLAP immunofluorescence in ChAT-positive neurons along the rostrocaudal extent of the nAmb (Fig. 5*B*), but not in the DMV (Fig. S5), indicating that our injections did not spread to the DMV.

**Figure 5.**
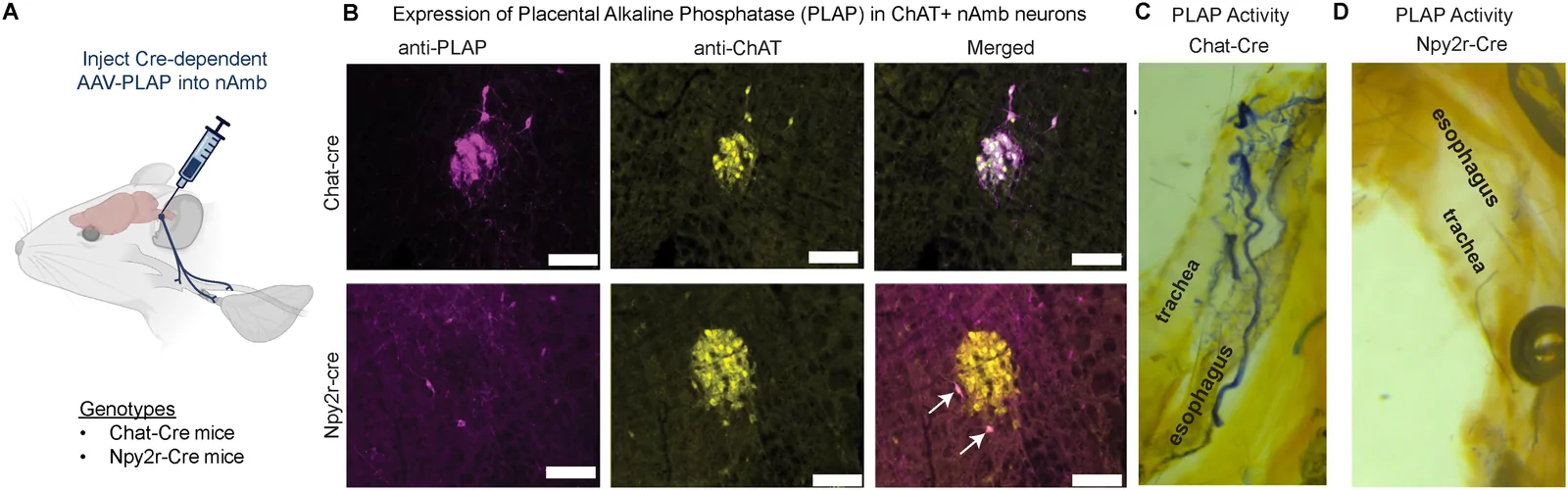
Npy2r^nAmb^ neurons do not innervate the upper airways or esophagus. ***A***, Strategy for targeting AAV-PLAP expression to Cre-expressing nAmb neurons of Chat-Cre and Npy2r-Cre mice. Figure panel created with Biorender.com. ***B***, PLAP and ChAT immunofluorescence in nAmb cells (*n* = 3 mice). Arrows indicate double-positive cells in an Npy2r-Cre mouse nAmb. Scale bar, 70 µm. ***C***, PLAP-stained axons from Chat^nAmb^ neurons in the upper airways and esophagus (*n* = 3 mice). ***D***, PLAP-stained axons from Npy2r^nAmb^ neurons in the upper airways and esophagus (*n* = 3 mice).

To visualize the axons of PLAP+ neurons, we chromogenically stained them while optically clearing the esophagus and upper airways as previously described (Prescott et al., 2020; Coverdell et al., 2022). Consistent with previous studies, we observed extensive PLAP-stained axons from Chat^nAmb^ neurons throughout the larynx, pharynx, and esophagus (*n* = 3 mice; representative image in Fig. 5*C*; Coverdell et al., 2022). However, we failed to observe any such projections from Npy2r^nAmb^ neurons (*n* = 3 mice; Fig. 5*D*, representative image). Together, our anterograde tracing studies indicate that Npy2r^nAmb^ neurons innervate the heart but not the upper airways or esophagus.

### Chemogenetically activating Npy2r^nAmb^ neurons lowers heart rate

Since Npy2r^nAmb^ neurons innervate the heart, we investigated whether they could control heart rate*.* To selectively activate these neurons, we relied on an intersectional chemogenetics approach, using an AAV which Cre- and Flp-dependently expresses the excitatory chemogenetic receptor hM3Dq (Strain et al., 2024). We first validated the specificity of this AAV by injecting it into the ventrolateral medulla of Chat-Cre;Phox2b-Flp mice, targeting nAmb neurons in general, based on their coexpression of *Chat* and *Phox2b* (Fig. S6*A*; Coverdell et al., 2022). We then quantified colocalization of immunofluorescence for ChAT and hM3Dq-HA in the nAmb, which is the only region where we observed hM3Dq-HA expression (data not shown). We observed that 91% of hM3Dq-HA+ cells were also ChAT+ in the nAmb, while 9% of hM3Dq-HA + nAmb cells had little to no ChAT immunofluorescence (*n* = 132 cells in total from six mice; Fig. S6*B*,*C*). The small percentage of hM3Dq-HA+/ChAT− cells most likely represents neurons with low ChAT expression, rather than Cre-independent (leaky) AAV transgene expression, since we failed to detect any hM3Dq-HA immunofluorescence after injecting this AAV into the nAmb of Phox2b-Flp mice that lacked Chat-Cre (Fig. S6*D*). These results confirm our previous finding that this AAV only expresses hM3Dq-HA in cells with both Cre and Flp recombinase activity (Strain et al., 2024).

Next, to selectively activate Npy2r^nAmb^ neurons while measuring heart rate, we injected the Cre- and Flp-dependent AAV-hM3Dq-HA into the ventrolateral medulla of Npy2r-Cre;Chat-Flp;R26^ds-HTB^ mice (Npy2r-Cre;Chat-Flp;hM3Dq^nAmb^ mice; Fig. 6*A*). This resulted in expression of hM3Dq-HA expression in a subset of nAmb neurons, mostly located in the external formation of the nAmb, where CVNs largely reside (Fig. 6*B*; Izzo et al., 1993). Of note, crossing Npy2r-Cre;Chat-Flp mice to the Cre/Flp reporter mouse strain, R26^ds-HTB^ (H2b-GFP; Bourane et al., 2015) resulted in H2b-GFP expression in much of the compact nAmb (Fig. 6*B*), where esophageal motor neurons reside (Fryscak et al., 1984; Bieger and Hopkins, 1987; Lee et al., 1992; Coverdell et al., 2022), which may be due to transient developmental expression of Npy2r-Cre in the compact nAmb (i.e., lineage labeling). In contrast, virally expressing hM3Dq-HA in adult Npy2r-Cre;Chat-Flp mice mostly labeled neurons in the external nAmb, with relatively few in the compact nAmb, consistent with both their cardiovagal identity and our results described above from virally infecting Npy2r-Cre nAmb neurons in the anterograde tracing studies.

**Figure 6.**
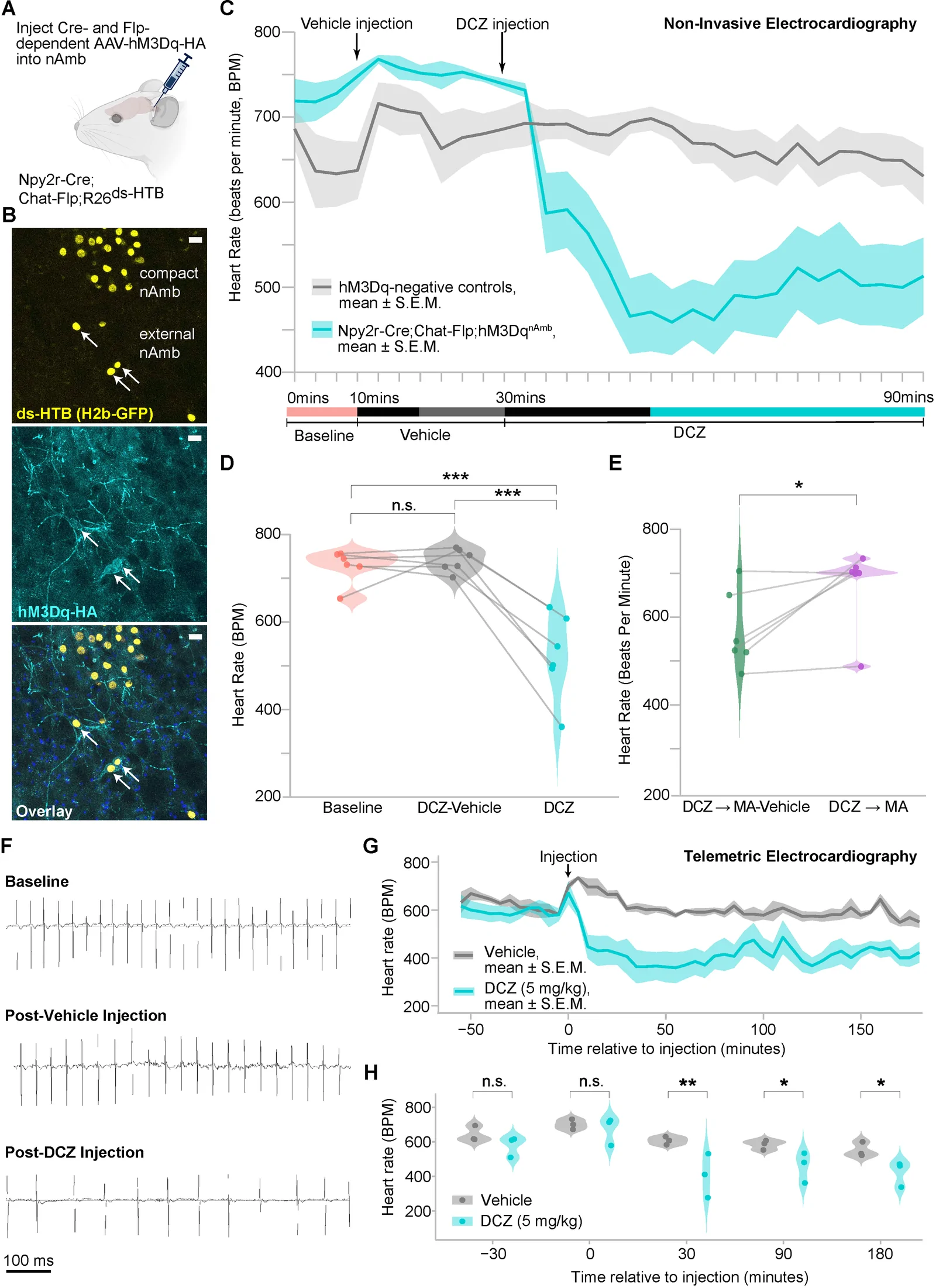
Chemogenetically activating Npy2r^nAmb^ neurons lowers heart rate. ***A***, Schematic of experimental design. Figure panel created with Biorender.com. ***B***, Representative image of H2b-GFP (R26^ds-HTB^ reporter) and hM3Dq-HA immunofluorescence in nAmb of Npy2r-Cre;Chat-Flp;R26^ds-HTB^ mice after injecting Cre- and Flp-dependent AAV-hM3Dq-HA into their ventrolateral medulla. Arrows indicate hM3Dq-HA-positive cells. The H2b-GFP observed in the compact nAmb is likely the result of transient developmental expression of Npy2r-Cre. ***C***, Heart rate measured in Npy2r-Cre;Chat-Flp;hM3Dq^nAmb^ mice by noninvasive ECG while chemogenetically activating Npy2r^nAmb^ neurons with the hM3Dq ligand DCZ (*n* = 6 mice). Heart rate was recorded every 3 min, with each line representing an individual measurement across time. For statistical analyses, heart rate values were averaged within each defined time window (baseline, vehicle, DCZ), and these window-averaged values were then used for all comparisons. The color-shaded parts of the timeline below the figure indicate the time windows used for averaging HR. ***D***, Administering DCZ (5 mg/kg, i.p.) significantly reduced heart rate in Npy2r-Cre;Chat-Flp;hM3Dq^nAmb^ mice relative to both baseline (HR_baseline_ = 728 ± 16 bpm; mean ± SD) and DCZ-vehicle treatment (HR_vehicle_ = 741 ± 11 bpm; mean ± SD). The reduction was observed for DCZ (HR_DCZ_ = 524 ± 40 bpm; mean ± SD) with an estimated mean difference of 204.35 ± 34.9 bpm compared with baseline (one-way repeated-measures ANOVA with Tukey's post hoc test, *p* = 5.8 × 10^−^⁴, *t*_(10)_ = 5.63) and 217.04 ± 36.3 bpm compared with vehicle (one-way repeated-measures ANOVA with Tukey’s post hoc test, *p* = 3.6 × 10^−^^4^, *t*_(10)_ = 5.98). ***p < 0.001. However, vehicle injection did not cause significant change in the heart rate compared with baseline (HR_baseline_, 728 ± 16 bpm; HRveh, 741 ± 11 bpm ; mean ± SD; Tukey post hoc following one-way repeated-measures ANOVA, *p* = 0.94). Colors correspond to the colored parts of the timeline below panel ***C***. ***E***, Administering MA bromide (1.0 mg/kg, i.p.), but not its vehicle (MA-veh), after DCZ rescued HR from bradycardia (HR_MA_ = 673 ± 37 bpm; HR_MA-veh_ = 569 ± 36 bpm; mean ± SD; estimated mean difference 103.64 ± 38.18 bpm, paired *t* test, *t*_(5)_ = −2.71, df = 5, *p* = 0.042). *p < 0.05. ***F***, ECG traces recorded telemetrically in Npy2r-Cre;Chat-Flp;hM3Dq^nAmb^ mice before and after injecting DCZ (or its vehicle) to chemogenetically activate Npy2r^nAmb^ neurons. Shown here are representative ECG traces from before DCZ injection (“baseline”; top trace), after DCZ-vehicle injection (middle trace), and after DCZ injection (bottom trace). Scale bar applies to all ECG traces. ***G***, With ECG telemetry, administering DCZ (5 mg/kg, i.p.) reduced heart rate in Npy2r-Cre;Chat-Flp;hM3Dq^nAmb^ mice relative to its vehicle (*n* = 3 mice). ***H***, With ECG telemetry, HR was significantly lower 30, 90, and 180 min after DCZ injection relative to vehicle injection in the same mice. A linear mixed-effects model with a Treatment (DCZ, Veh) by Time (−30, 0, 30, 90, 180) interaction and a random effect of mouse found a significant main effect of Treatment (*F*_(1,20)_ = 19.56, *p* < 0.001) and Time (*F*_(4,20)_ = 9.27, *p* < 0.001), but not the interaction (*F*_(4,20)_ = 1.43, *p* = 0.258). Post hoc testing with a Holm adjustment at time points −30, 0, 30, 90, and 180 min revealed that DCZ significantly decreased HR in comparison with vehicle at 30 (Veh–DCZ = 200 bpm, *t*_(18)_ = 3.638, *p* = 0.0019), 90 (Veh–DCZ = 124 bpm, *t*_(18)_ = 2.258, *p* = 0.0366) and 180 (Veh–DCZ = 128 bpm, *t*_(18)_ = 2.324, *p* = 0.032) minutes after injection. HR at −30 and 0 min postinjection was not significantly different between Veh and DCZ (both *p* > 0.05).

We next measured heart rate (HR) noninvasively in awake mice (Strain et al., 2024) while chemogenetically activating their Npy2r^nAmb^ neurons with a nonmetabolizable ligand for hM3Dq, DCZ (Nagai et al., 2020). After acclimation to the ECG system, each mouse underwent a series of intraperitoneal injections while we recorded its HR (*n* = 6 mice). Injecting the vehicle for DCZ into Npy2r-Cre;Chat-Flp;hM3Dq^nAmb^ mice did not significantly alter HR from baseline (HR_baseline_, 728 ± 16 bpm; HR_veh,_ 741 ± 11 bpm_;_ mean ± SD). Subsequent injection of DCZ (5 mg/kg, i.p.), however, significantly lowered HR to 524 ± 40 bpm compared with both vehicle and baseline levels (Fig. 6*C*,*D*). However, injecting the same dose of DCZ into wild-type mice did not significantly alter their HR relative to either baseline or DCZ-vehicle injection (2% DMSO in 0.9% sterile saline), indicating that DCZ on its own does not affect HR (*n* = 6 mice; Fig. 6*C*; Fig. S6*E*). In addition, a dose–response curve in Npy2r-Cre;Chat-Flp;hM3Dq^nAmb^ mice showed that lower doses of DCZ, including 0.01 mg/kg and 0.1 mg/kg, also significantly reduced HR when compared with vehicle injections (Fig. S6*F*). Our results thus indicate that chemogenetically activating Npy2r^nAmb^ neurons can robustly decrease HR. Subsequent injection of DCZ (5 mg/kg, i.p.).

The vagus nerve decreases HR through a canonical downstream signaling pathway in which postganglionic cardiac neurons inhibit SA node myocytes by activating muscarinic ACh receptors. To determine whether Npy2r^nAmb^ neurons use this signaling pathway to decrease HR, we administered MA (1.0 mg/kg, i.p.), a peripherally restricted muscarinic receptor antagonist. Mice were administered DCZ (5 mg/kg, i.p.) and then either MA or its vehicle in separate trials while we recorded their HR (*n* = 6 mice). Administering MA, but not its vehicle, after DCZ significantly increased HR (Fig. 6*E*), rescuing HR from the DCZ-induced bradycardia. Thus, activating Npy2r^nAmb^ neurons decreases HR through a canonical cardiovagal signaling pathway involving peripheral muscarinic receptors.

One limitation of our noninvasive ECG method is that the recordings are intermittent and require repeated transfers of mice between their home cages and the ECG system for long-term recording. Therefore, we cannot effectively assess whether changes in cardiac activity are sustained over minutes to hours. For this reason, we repeated our chemogenetic studies in a separate cohort of Npy2r-Cre;Chat-Flp;hM3Dq^nAmb^ mice in which we first implanted radio telemetry probes for continuous ECG (*n* = 3 mice). After recovery and acclimation to the ECG telemetry system, we recorded baseline HR at a sampling rate of 1,000 Hz while the mice remained in their home cages for ∼1 h. We then injected either DCZ (5 mg/kg, i.p.) or its vehicle, placed the mice back into their home cages, and recorded HR continuously for 3 h. After injecting the mice with DCZ, we observed less frequent QRS complexes than before injection (baseline) or after vehicle injection, though the ECG waveform was qualitatively similar across conditions (Fig. 6*F*). Similar to our results with noninvasive ECG, we observed a decrease in HR after injecting the mice with DCZ but not its vehicle (Fig. 6*G*). Comparing 5 min averages at different timepoints revealed that HR was significantly lower 30, 90, and 180 min after DCZ injection, relative to vehicle injection (Fig. 6*H*). Thus, our results from noninvasive and telemetric ECG together demonstrate that chemogenetically activating Npy2r^nAmb^ neurons robustly decreases HR.

### Voluntary underwater diving activates Npy2r^nAmb^ neurons

Voluntary underwater submersion elicits the diving reflex in mammals, including a powerful vagal bradycardia (Panneton, 2013). Based on the bradycardic response we observed when chemogenetically activating Npy2r^nAmb^ neurons, we hypothesized that these neurons would be activated during the diving reflex. We tested this hypothesis by evaluating expression of the immediate early gene and neuronal activation marker, *Fos*, in nAmb neurons after voluntary underwater diving.

We trained mice to voluntarily dive up to 20 cm underwater to reach an escape platform (Fig. 7*A*). We validated that this model evokes a diving response by measuring ECG with radio telemetry implants (Fig. 7*B*). Heart rate decreased markedly upon initiation of the dive and remained low while underwater before recovering to baseline, following a brief overshoot after the mice resurfaced (*n* = 10 mice; Fig. 7*A*,*B*), consistent with previous descriptions (Hult et al., 2019). We then performed a session of repeated diving for up to 30 mins to robustly activate the diving response before perfusing mice for histology. Controls were handled similarly and allowed to explore the test chamber with a shallow water level but did not perform any dives (Fig. 7*C*). In mice that performed voluntary diving, we observed a 174% increase in the proportion of Npy2r^nAmb^ neurons that expressed *Fos* (*n* = 6–7 mice; dive vs control, 44.5 ± 5.5% vs 16.3 ± 4.3% Npy2r^nAmb^ neurons expressing Fos, unpaired *t* test, *t* = 3.915, df = 11, *p* = 0.0024; Fig. 7*D*–*F*). Cell counts for total Npy2r^nAmb^ neurons were similar between cases (42 ± 6 vs 45 ± 6 cells per unilateral nAmb in a one-in-three series, unpaired *t* test, *t* = 0.3679, df = 11, *p* = 0.72), and a similar proportion of FG cells expressed Npy2r between cases (17.6 ± 1.6 vs 20.6 ± 3.5% of all FG cells expressed *Npy2r*, unpaired *t* test, *t* = 0.7933, df = 11, *p* = 0.44). These data indicate that Npy2r^nAmb^ neurons are activated during voluntary diving, consistent with the role of these neurons in the parasympathetic control of heart rate.

**Figure 7.**
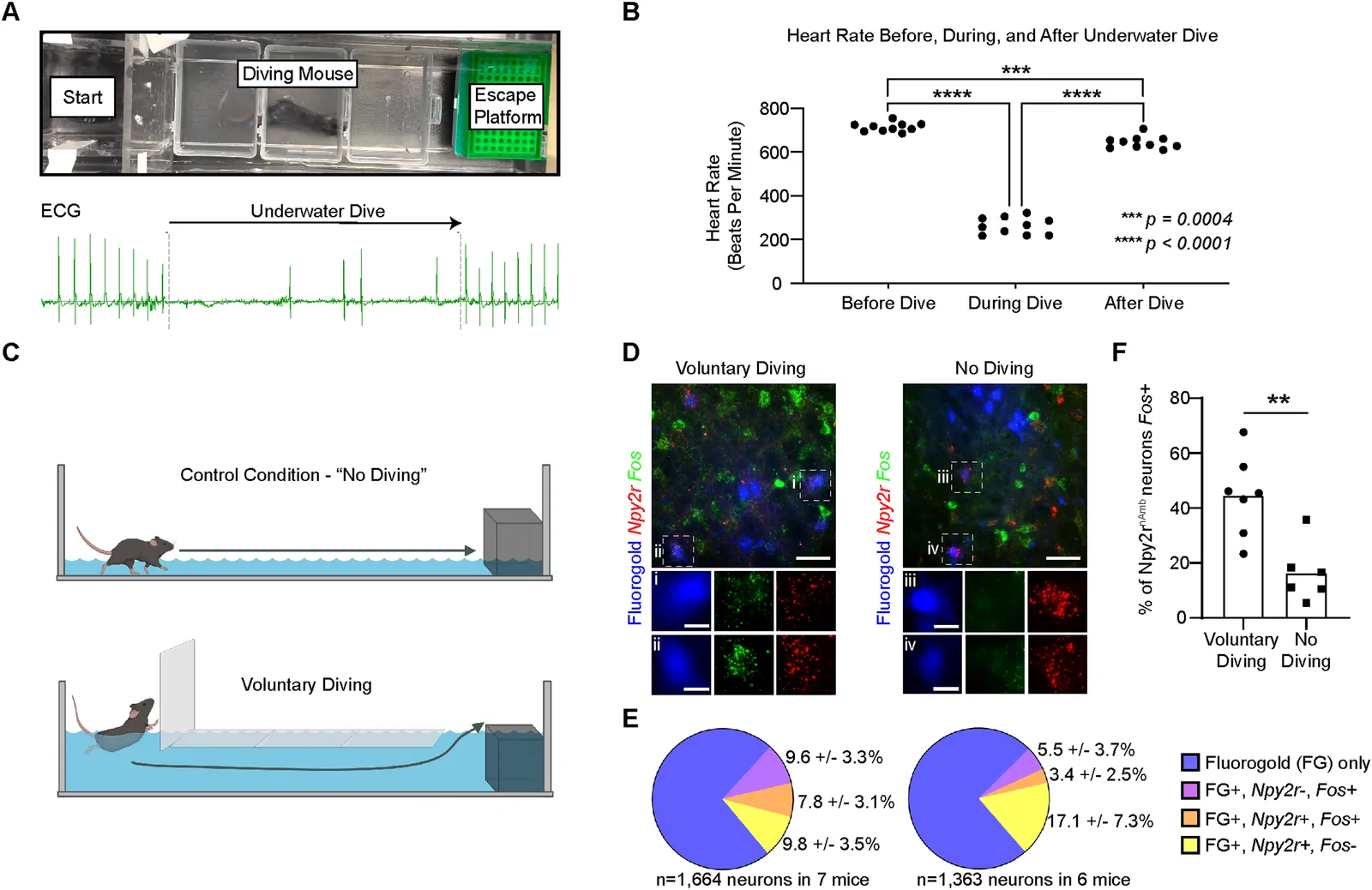
Voluntary underwater diving activates Npy2r^nAmb^ neurons. ***A***, Experimental setup for voluntary diving assay (top panel). ECG recorded during a voluntary dive (bottom panel). ECG signal was collected using an implanted radio telemetry device. ***B***, Grouped data for heart rate during voluntary diving. ***C***, Experimental design to compare *Fos* expression in Npy2r nAmb neurons during voluntary diving and no-diving controls. Trained mice performed ∼20–40 dives over a 20–30 min period followed by 30 min recovery period before perfusion for histology. Mice in the control condition performed no dives but were subjected to similar handling procedures. Figure panel created with Biorender.com. ***D***, RNA FISH of the immediate early gene *Fos* and the nAmb neuron subtype marker *Npy2r* after voluntary diving and control conditions. Systemically administered Fluorogold labels all neurons that project outside the central nervous system, including all vagal efferent neurons in the nAmb. Scale bars, 100 μm for large, overlay images and 25 μm for small, single-color images. ***E***, Quantification of *Fos* and *Npy2r* expression in Fluorogold + nAmb neurons during voluntary diving (left) and control (right) conditions. ***G***, Comparison of the proportion of *Npy2r* + nAmb neurons that expressed *Fos* in the voluntary diving and control condition. Unpaired *t* test, *t* = 3.915, df = 11, *p* = 0.002.

## Discussion

This study identified cardiovagal nAmb neurons based on their molecular, anatomical, and functional features. Transcriptomic profiling and clustering analysis of 1,245 *Chat-*expressing neurons in the ventrolateral medulla indicated nine molecular subtypes. RNA FISH showed that *Adcyap1* + nAmb neurons and *Npy2r* + nAmb (Npy2r^nAmb^) neurons are rare and partially overlapping populations of nAmb neurons. Activating Npy2r^nAmb^ neurons with intersectional chemogenetics significantly decreased heart rate through a canonical cardiovagal signaling pathway. Finally, voluntary underwater diving significantly increased the percentage of *Fos*-expressing Npy2r^nAmb^ neurons, indicating that these neurons are activated during the diving reflex. Overall, our study identifies Npy2r^nAmb^ neurons as a molecular subtype which innervates cardiac ganglia but not the esophagus or upper airways, is activated during the diving reflex, and decreases heart rate upon activation.

Our study provides a more comprehensive understanding of the molecular subtypes of nAmb neurons than previously known. Two studies previously used single-cell RNA-seq to molecularly classify subtypes of nAmb neurons. For instance, Coverdell et al. (2022) identified three molecular subtypes based on profiling gene expression in 145 *Chat*-expressing nAmb neurons of adult mice (Coverdell et al., 2022). Another study, Veerakumar et al. (2022), classified three subtypes of nAmb neurons from 203 heart- or larynx-projecting nAmb neurons of neonatal mice (Veerakumar et al., 2022). However, each of these previous studies contained far fewer neurons than the nearly 1,200 found bilaterally in adult mouse nAmb (Sturrock, 1990) and so may have underestimated the diversity of nAmb neurons. Our present study identified five molecular subtypes of nAmb neurons and one molecular subtype of intermediate-zone CVNs among 1,245 Chat-expressing cells, nearly doubling the number of previously recognized nAmb neuron subtypes. However, our results do not exclude the presence of rare nAmb neuron subtypes. Moreover, it remains unclear whether the composition of subtypes differs between the left and right hemispheres of the nAmb or varies with sex, particularly given that the right vagus controls heart rate more strongly than the left (Muppidi et al., 2011).

Our present study also provides a finer resolution of nAmb neuron subtypes than previous studies. For instance, a previous study by our group identified three subtypes of nAmb neurons (Coverdell et al., 2024), which our integrated analysis now resolves as six subtypes. One subtype identified in our previous dataset, Adcyap1^nAmb^ neurons, maps to multiple subtypes in the integrated dataset. Another previously defined subtype, Vipr2^nAmb^ neurons, maps to two subtypes, n05 (Uts2^nAmb^) and n06 (Stk32a^nAmb^), in the present study. Since Vipr2^nAmb^ neurons innervate the pharynx and larynx, future studies could investigate whether the Uts2^nAmb^ and Stk32a^nAmb^ subtypes separately innervate these two upper airway regions. Interestingly, *Uts2* and *Uts2b* expression mark vocal control regions in birds (Bell et al., 2019) and is enriched in the n05/Uts2^nAmb^ cluster of mouse nAmb neurons in the current study, raising the possibility that this subtype innervates the larynx to control vocalization.

Comparing the molecular subtypes of nAmb neurons in the present study to those of a previous study also sheds light on the functional identities of our nAmb neuron subtypes. Veerakumar and colleagues identified three functional subtypes of nAmb neurons: cardiopulmonary nAmb neurons (ACP), which cause bradycardia and bronchoconstriction; cardiovascular nAmb neurons (ACV), which cause bradycardia without bronchoconstriction; and larynx-projecting nAmb neurons (Veerakumar et al., 2022). Marker gene expression between the current study and Veerakumar et al. suggests that clusters n03.s01 (Npy2r^nAmb^ neurons) and n03.s02 of the current study may correspond to ACV neurons (*Bche+*) and ACP (*Calb1+*) neurons, respectively, of the Veerakumar et al. study. However, differences in gene expression between the datasets, potentially related to age differences in mice, limit our ability to compare nAmb neuron subtypes between the two studies. In addition, our anterograde tracing studies did not rule out the possibility that Npy2r^nAmb^ neurons also innervate the lungs and so cannot confirm anatomically that they are ACV neurons.

The activation of Npy2r^nAmb^ neurons during underwater diving is consistent with a well-characterized trigemino-cardiac reflex circuit. Nasal sensory input from the anterior ethmoidal nerve engages neurons in the medullary dorsal horn which then relay to the rostral ventrolateral medulla (RVLM), where cardiovagal preganglionic neurons lie within the external formation of the nAmb (Thomas and Calaresu, 1974; Ciriello and Calaresu, 1982; Panneton et al., 2012; Panneton et al., 2014; Panneton and Gan, 2020). Activation of trigeminal afferents evokes a polysynaptic excitatory glutamatergic pathway to CVNs of nAmb (Gorini et al., 2009; Gorini et al., 2010), confirming that these neurons serve as direct functional targets of trigemino-cardiac signaling. Together, anatomical tracing, physiological stimulation, and lesion studies point to a short, robust reflex circuit with both direct and indirect pathways from trigeminal sensory neurons to cardiac efferent neurons. These mechanisms provide a framework for understanding how somatosensory stimuli engage parasympathetic output and may help inform therapeutic use of the diving response.

Importantly, while we observed increased *Fos* expression in Npy2r^nAmb^ neurons during voluntary diving, this does not establish the role of these neurons in diving-induced bradycardia. Loss-of-function experiments, such as inhibiting or ablating Npy2r^nAmb^ neurons during voluntary diving, will be necessary to determine the contribution of Npy2r + nAmb neurons to this reflex.

Our retrograde and anterograde tracing studies revealed that Npy2r^nAmb^ neurons innervate cardiac ganglia but not the esophagus and upper airways. However, these studies have several limitations. One limitation is that our retrograde Fluorogold injections into the pericardiac fat pad likely do not label all CVNs. In addition, though this approach reliably labels heart-projecting neurons, it does not distinguish between neurons innervating specific cardiac targets, such as the nodes, ventricles, or intrinsic cardiac ganglia. Another limitation is that our anterograde tracing studies do not quantify the number or location of cardiac ganglia innervated by Npy2r^nAmb^ neurons. Thus, our retrograde and anterograde tracing studies identified CVNs only qualitatively, based on their innervation of the heart. Additional studies are needed to characterize the functional roles of these molecularly distinct subtypes of CVNs, determine which fraction of each subtype innervates the heart, and quantitatively map their innervation of the heart. Finally, while we examined the pericardiac fat pad to assess the spread of the Fluorogold injection, we cannot rule out the possibility that some Fluorogold leaked from the injection site and labeled noncardiovagal nAmb neurons projecting nearby. However, our anterograde tracing results do confirm that at least some Npy2r^nAmb^ neurons innervate the heart.

Finally, the molecular profile of Npy2r + nAmb neurons raises hypotheses about signaling pathways through which the vagus nerve controls heart rate. Our analysis identified several genes enriched in this cluster that mediates vagal control of heart rate. For instance, *Prkg1,* which encodes cGMP-dependent protein kinase I, acts as a key effector in nitric oxide (NO) signaling and has been implicated in modulating cardiac contractility and autonomic control of heart rate via NO-cGMP pathways (Layland et al., 2002). Similarly, *Adcy8*, a calcium-sensitive adenylyl cyclase, has been shown to affect heart rate and heart rate variability when overexpressed (Moen et al., 2019). *Ank2*, encoding ankyrin-B, plays a crucial role in targeting cardiac ion channels, transporters, and signaling proteins to the membrane; loss-of-function mutations in *Ank2* can lead to a spectrum of arrhythmias, including sinus node dysfunction, atrial fibrillation, and ventricular arrhythmias, collectively known as “ankyrin-B syndrome” (Huq et al., 2017). Additionally, *Dmd*, which encodes dystrophin, is critical for cardiac integrity, as its deficiency underlies Duchenne cardiomyopathy, characterized by fibrosis, arrhythmias, and heart failure (Schultz et al., 2022). Future studies using conditional knockouts could investigate whether these genes control heart rate through their action in Npy2r^nAmb^ neurons.

## Data Availability

The raw and processed snRNA-seq data and metadata reported in this paper are available at GEO accession number GSE348348. A user-friendly interface for visualizing and exploring the fully processed snRNA-seq data is available at the Broad Institute Single Cell Portal at https://singlecell.broadinstitute.org/single_cell/study/SCP3861/molecular-disambiguation-of-heart-rate-control-by-the-nucleus-ambiguus. The code used for processing, clustering, and visualizing the clustered snRNA-seq data and the clustering data files are available through Zenodo at https://zenodo.org/records/22864368. Any additional information required to reanalyze the data reported in this paper is available from the lead contact upon reasonable request.
